# Exosomal circZNF800 Derived from Glioma Stem-like Cells Regulates Glioblastoma Tumorigenicity via the PIEZO1/Akt Axis

**DOI:** 10.1007/s12035-024-04002-0

**Published:** 2024-02-07

**Authors:** Ning Zhang, Pengfei Wu, Maolin Mu, Chaoshi Niu, Shanshan Hu

**Affiliations:** 1 Department of Neurosurgery, Division of Life Sciences and Medicine, The First Affiliated Hospital of USTC, University of Science and Technology of China, Hefei, Anhui 230001 People’s Republic of China; 2 Anhui Key Laboratory of Brain Function and Diseases, Hefei, Anhui 230001 People’s Republic of China; 3 Anhui Provincial Stereotactic Neurosurgical Institute, Hefei, Anhui 230001 People’s Republic of China; 4 Anhui Provincial Clinical Research Center for Neurosurgical Disease, Hefei, Anhui 230001 People’s Republic of China

**Keywords:** Glioma stem-like cell, Exosome, circZNF800, miR-139-5p, PIEZO1

## Abstract

Exosomes play a crucial role in regulating crosstalk between tumor and tumor stem-like cells through their cargo molecules. Circular RNAs (circRNAs) have recently been demonstrated to be critical factors in tumorigenesis. This study focuses on the molecular mechanism by which circRNAs from glioma stem-like cell (GSLC) exosomes regulate glioblastoma (GBM) tumorigenicity. In this study, we validated that GSLC exosomes accelerated the malignant phenotype of GBM. Subsequently, we found that circZNF800 was highly expressed in GSLC exosomes and was negatively associated with GBM patients. CircZNF800 promoted GBM cell proliferation and migration and inhibited GBM cell apoptosis in vitro. Silencing circZNF800 could improve the GBM xenograft model survival rate. Mechanistic studies revealed that circZNF800 activated the PIEZO1/Akt signaling pathway by sponging miR-139-5p. CircZNF800 derived from GSLC exosomes promoted GBM cell tumorigenicity and predicted poor prognosis in GBM patients. CircZNF800 has the potential to serve as a promising target for further therapeutic exploration.

**Supplementary Information:**

The online version contains supplementary material available at 10.1007/s12035-024-04002-0.

## Introduction

Glioma is the most aggressive primary tumor of the central nervous system (CNS) with a poor prognosis [1]. GBM is determined by the World Health Organization (WHO) to be the highest grade of glioma and presents as a diffuse tumor with invasion into the normal brain [2–4]. Many studies have demonstrated that a group of GBM cells with self-renewal ability and differentiation potential are GSLCs [5–7]. GSLCs are thought to underlie GBM initiation and provide the driving force for GBM growth and maintenance [8, 9].

Exosomes are small extracellular vesicles (EVs) with a diameter of 40 to 160 nm (average ~ 100 nm) that mediate communication between cells [10, 11]. Exosomes contain constituents such as proteins, lipids, DNA, mRNAs and noncoding RNAs, which play an important role in tumor angiogenesis, invasion, metastasis, and drug resistance [11–13].

CircRNAs are a new class of RNA transcripts that are produced from head-to-tail splicing of exons and are different from mRNAs in their production and structure, therefore they have unique cellular functions and potential biomedical applications [14]. Accumulating evidence shows that circRNAs act as important regulators in cancers, including GBM [13, 15, 16]. The important functions and mechanisms of circRNAs have been identified: (1) circRNA acts as a miRNA sponge [14, 17]; (2) circRNA functions as sponges or decoys for proteins and indirectly regulates their functions [13, 18]; (3) circRNA regulates transcription by interacting with small nuclear ribonucleoproteins (snRNPs) [19]; and (4) circRNA can be translated through internal ribosome entry site (IRESs) and N^6^-methyladenosine (m^6^A)-mediated cap-independent translation initiation [20, 21].

In addition, microRNAs (miRNAs) (∼21 to 23 nucleotides in length) are widely expressed in species. A number of researches revealed that miRNAs participate in regulating tumor proliferation, metastasis, and metabolism by binding to the 3'untranslated region (3'UTR) of target mRNAs [22–26].

Currently, there are few reports describing the role of GSLC-exosomal circRNAs in GBM. The biological functions of circRNAs in GBM are not fully understood and require further investigation. In this study, we performed high-throughput RNA sequencing (RNA-seq) of exosomes derived from GSLCs and HEB cells (human normal astrocytes were used to be the control in cell lines). Subsequently, we identified a novel circular RNA (hsa_circ_0082096) named circZNF800, which is highly expressed in GSLC exosomes, and found that circZNF800 regulated GBM cells proliferation, migration, and apoptosis in vitro. Furthermore, mouse models were used to clarify the biological function of circZNF800 in GBM tumorigenesis. Mechanistically, we demonstrated that circZNF800 affected the PIEZO1/AKT axis by sponging miR-139-5p and ultimately acted as a tumor promoter in GBM.

## Methods

### Patients and Specimens

All glioblastoma patient tumor samples and normal tissues were collected from The First Affiliated Hospital of University of Science and Technology of China, which were approved by the Human Research Ethics Committee of the hospital. In this study, we obtained written informed consent from each patient. A total of 31 resected glioblastoma tumors were obtained from June 2019 to January 2022, and all tumor tissues were clinically and histopathologically diagnosed as glioblastoma. Normal brain tissues were obtained from 15 patients with brain tissue resection due to craniocerebral injury during the period from June 2019 to January 2022. All samples were rinsed with PBS after the operation and cut into small pieces. All samples were stored at -80 ℃ for the following experiments. The information of all GBM patients was listed in Table 1.

**Table 1:** The relationship of circZNF800 and clinical characteristics in 31 glioma patients

Variable		circZNF800	
		High (n = 16)	Low (n = 15)
Sex	Male Female	11 5	9 6
Age(year)	≤ 45 > 45	2 14	3 12
Location	Frontal Parietal Occipital Temporal	3 2 7 4	3 3 6 3
Recurrence	NO Yes	2 14	2 13

### Cell Lines and Cell Culture

Human glioblastoma (GBM) U87, U251 cell lines, normal human astrocyte cell line (HEB) and 293 T cell lines were purchased from the American Type Culture Collection (ATCC). These cells were authenticated using an STR assay (BGI, China). All cells were cultured in Dulbecco's modified Eagle's medium (HyClone, China) with 10% fetal bovine serum (Clark Bioscience, USA) and 1% penicillin–streptomycin-amphotericin B solution (Solarbio, China) at 37 °C in a humidified atmosphere with 5% CO_2_. The glioma stem-like cells (GSLCs) were purified from the neurospheres by magnetic cell sorting with CD133 microbeads (Miltenyi Biotec, Germany). The GSLCs were cultured with DMEM/F12 serum-free medium supplemented with 2% B27 (Gibco, USA), 20 ng/mL bFGF (PeproTech, USA) and 20 ng/mL EGF (Gibco, USA) in a non-adhesive culture system.

### Exosome Purification and Characterization

Debris and dead cells in the medium were removed by centrifugation at 3000 × g for 30 min and then filtered through a 0.22 μm filter. The medium was then subjected to 120,000 × g for 70 min at 4 °C. After washing with PBS (120,000 × g for 70 min), the exosomes were resuspended in PBS. The morphology of exosomes was characterized by transmission electron microscopy (TEM) (Hitachi H-7650, Japan). The characterization of exosomes was confirmed by measuring the expression of exosome-specific markers and by western blotting and particle size by NanoSight analysis. To monitor exosome trafficking, exosomes were labeled with PKH26 (Sigma-Aldrich, USA). After PKH26 staining, the exosomes were washed in PBS and collected by ultracentrifugation (120,000 × g for 30 min) at 4 °C. PKH26-labeled exosomes were resuspended in PBS.

### RNA Extraction and Quantitative Reverse Transcriptase PCR (qRT-PCR)

Total RNA was extracted by using TRIzol (Invitrogen, USA) according to the manufacturer's instructions. RNase R treatment was carried out for 30 min at 37 °C using 3 U/mg of RNase R (Epicenter Technologies, USA). Dzup Reagent (Sangon, China) was used to extract gDNA. Sample cDNA was synthesized using the GoScript Reverse Transcription System (Promega, USA) according to the manufacturer's protocol. Quantitative real-time PCR was performed with TransStart Green qPCR SuperMix (TransGen, China) on a real-time PCR system (Roche LightCycler96, Germany). GAPDH or U6 was used as the internal control, and the relative expression of target genes was calculated by the 2^−ΔΔCt^ method. Sequences of the primers used for qRT-PCR in this study are listed in Table S1.

### Western Blotting

For western blotting, cells were placed on ice and washed twice with PBS. Proteins were extracted with RIPA buffer (Beyotime, China) plus phenylmethylsulfonyl fluoride and phosphatase inhibitor cocktail (Beyotime, China). Samples were loaded per well on 4%-15% or 10% SDS-PAGE gels and transferred onto PVDF membranes (Millipore, USA) activated by methanol. Membranes were washed with TBST, blocked with 5% nonfat milk and incubated with antibodies against GAPDH (1:2000, Sangon, D190090-0100, China), CD9 (1:1000, Proteintech, 60,232–1-Ig, China), CD63 (1:1000, Proteintech, 67,605–1-Ig, China), PIEZO1 (1:2000, Proteintech, 28,511–1-AP, China), FAK (1:2000, Proteintech, 66,258–1-Ig, China), p-FAK (1:2000, Cell Signaling Technology, 8556 T, USA), Akt (1:2000, Proteintech, 10,176–2-AP, China) and p-Akt (1:2000, Cell Signaling Technology, 4060S, USA). Secondary antibodies (1:5000, goat anti-rabbit IgG-HRP and goat anti-mouse IgG-HRP, Sangon, China) and the protein bands were visualized using a chemiluminescence reagent ECL kit (Thermo Fisher, USA).

### Cell Viability Assay

Cell viability was assessed by CCK-8 assays (Biosharp, China). Cells were seeded in 96-well plates (8 × 10^3^ cells per well). The absorbance of each sample was read at a wavelength of 450 nm on an Infinite M200 (Tecan, Switzerland).

### Cell Migration Assay

Cell migration ability was assessed by Transwell assays. Cells (1 × 10^4^ cells per well) were seeded in the upper chambers (Corning, USA) in serum-free media without the Matrigel membrane. Meanwhile, the lower chambers were loaded with 10% FBS. After incubation at 37 °C and 5% CO_2_ for 48 h, the upper chamber was cleaned with a cotton swab, and the lower chamber was washed with PBS, fixed with 4% paraformaldehyde, stained with 0.1% crystal violet (Sangon, China) for 20 min, and washed with PBS. The lower chamber was imaged by an inversion microscope (Olympus, Japan).

### Cell Apoptosis Assay

Cell apoptosis was assessed by flow cytometry. Cells (5 × 10^5^ cells/well) were seeded in a six-well plate. After treatment, cells were harvested by centrifugation at 2000 rpm for 5 min, washed with PBS three times, and then incubated with 5 μL of FITC-conjugated Annexin V and 5 μL of PI for 15 min at room temperature in the dark. The stained cells were detected by BD FACS flow cytometer (BD Biosciences, USA).

### Co-Culture Assay

Glioma stem-like cells were placed in the upper chamber and glioblastoma cells in the lower chamber where cells were co-cultured at a ratio of 1:1 using a trans-well plate (0.4 mm polycarbonate filter, Corning, USA) for 24 h. To inhibit extracellular vesicle secretion, glioma stem-like cells were pretreated with GW4869 (an inhibitor of neutral sphingomyelinase, 10 μM, Sigma-Aldrich, USA).

### RNA FISH

RNA FISH analysis of the GBM cell lines was performed using an RNA FISH kit (RiboBio, China) according to the manufacturer’s protocols. Briefly, U87 and U251 cells were allowed to grow until 70–80% confluency. The cells were fixed in 4% formaldehyde for 10 min, washed 3 times with PBS for 5 min, permeabilized with 0.5% Triton-X-100 in PBS for 15 min at 4 °C, and washed with PBS 3 × 5 min. The cells were incubated with a Cy3-labeled circZNF800 probes mixture at 37 °C overnight and washed with prewarmed 2 × saline-sodium citrate (SSC) 5 times for 3 min. DNA was stained with DAPI and visualized using an Olympus camera.

### Plasmid Construction and Cell Transfection

To construct the circZNF800 overexpression vector, the second and third exons of ZNF800 gene and the endogenous flanking sequence including the complementary Alu element pairs were inserted into the backbone vector of pcDNA3.0, whereas the mock vector with no circZNF800 sequence was used as a control. Plasmid, siRNA (Ribobio, China), miRNA mimic (Hanbio, China) and miRNA inhibitor (Hanbio, China) were conducted with Lipofectamine 8000 (Beyotime, China) according to the manufacturer's protocol.

### Subcellular Fractionation

Nuclear and cytoplasmic separation was performed using the Nuclei Isolation Kit (Keygenbio, China) according to the manufacturer's instructions.

### RNA Pull-Down Assay

For RNA pulldown assay, cells were washed with cold PBS and then cross-linked in the UV cross-linker (UVP CL-1000, USA). The cells were scraped and resuspended in RIPA buffer (50 mM Tris–HCl, pH 8.0, 150 mM NaCl, 5 mM EDTA, 1% NP-40, 0.1% SDS, 1 mM DTT, complete protease inhibitor and 0.1 U/µL RNase inhibitor) for 10 min on ice and then harvested and sonicated for 15 min. Then, the samples were centrifuged at 13,000 rpm for 20 min. Then, 100 pmol 5'-biotinylated probes were added to the supernatant at 4 °C for 2 h. Streptavidin Dynabeads beads (M-280, Invitrogen, USA) were washed three times with RIPA buffer and supplemented with 1 mg/ml BSA and 0.5 mg/mL yeast total RNA rotated for 1 h. The blocked beads were added to the supernatant with probes and then rotated for 4 h at 4 °C. After washing three times with RIPA buffer supplemented with 500 mM RIPA buffer, beads were harvested with magnets.

### RNA Immunoprecipitation

The cell (4 × 10^6^) supernatant was discarded after centrifugation at 1000 × g at 4 °C for 5 min. The cells were washed with precooled PBS twice, and the supernatant was discarded. The cells were suspended with lysate and gently blown away for an ice bath for 10 min. The protein-A/G-coated magnetic beads were fully suspended, and then 75 μL beads were added to 1.5 mL EP tubes. After NT-2 buffer washing twice, resuspension with 100 μL NT-2 buffer, add 5 μg AGO2 or IgG antibody, and mix at room temperature for 1 h. Centrifuge 5000 × g for 15 s, add magnets to absorb magnetic beads, and remove supernatant. The tube was removed from the magnets, and 1 mL NT-2 was added. The tube was blown and mixed well and centrifuged at 5000 × g for 15 s, which was repeated 3 times. Samples were resuspended in 900 μL NT-2 buffer. Cell lysates were centrifuged at 20,000 × g at 4 °C for 10 min. Then, 100 μL supernatant was added to the prepared magnetic beads suspended in 900 μL NT-2 buffer and incubated with the antibody. A 10 μL sample was used as input. Vertical mixing was performed at 4 °C for more than 3 h or overnight. The sample was briefly centrifuged to the bottom of the tube, and the supernatant was discarded, which was repeated 3 times. Precipitation is the sample obtained in the RIP experiment, and RNA can be further extracted after digestion by 0.5 mg/ml Proteinase K (Beyotime, China) for subsequent analysis.

### Dual Luciferase Reporter Assay

The StarBase (http://starbase.sysu.edu.cn/) or TargetScan (http://www.targetscan.org) databases were used to predict the potential binding site. CircZNF800 or PIEZO1 fragments containing predicted wild-type (wt) or mutant (mut) miR-139-5p binding sites were cloned into pcDNA3.0. 293 T cells were cultured in 24-well plates and transfected with 200 ng luciferase reporter plasmids (containing wild-type or mutant) using Lipofectamine 8000 (Beyotime, China). Relative luciferase activity was measured 48 h after transfection using a dual luciferase reporter assay system (Promega, USA) according to the manufacturer's instructions. Firefly luciferase activity was normalized to the corresponding Renilla luciferase.

### Fluorescence Imaging of Ca2+ Levels with a Confocal Microscope

Ca^2+^ levels were assessed using a Ca^2+^ fluorescence indicator fluo-3-AM (absin, China). Cells were incubated with 4 μM fluo-3-AM at 37 °C 30 min. Fluorescent dye-loaded cells were washed with PBS at room temperature. Continuously, cells were staibed with DAPI. Fluo-3-AM loaded cells were excited with a confocal microscope at wavelength of 488 nm and the data were collected for emission intensity at a wavelength of 515 nm.

### Xenograft Model in Vivo

Four female BALB/c mice (5 weeks) obtained from GemPharmatech (China) were maintained in pathogen-free conditions. Intracranial tumorigenesis in nude mice with control and sh-circZNF800 U251 luciferase cells (5 × 10^5^) (*n* = 4/per group). The bioluminescence was monitored every 6 days. The representative bioluminescence imaging of metastases was measured by a spectrum luminal imager (IVIS Spectrum, USA).

### Immunohistochemistry (IHC)

The transplanted tumor model tissues were fixed with 4% paraformaldehyde, embedded in paraffin, and cut into 4 μm sections. The sections were then treated with xylene and ethanol to remove paraffin. After blocking with 5% normal goat serum, sections were treated with anti-PIEZO1 (1:200, Proteintech, 28,511–1-AP, China), anti-FAK (1:200, Proteintech, 66,258–1-Ig, China), anti-pFAK (1:200, Thermo Fisher Scientific, PA5-17,084, USA), anti-Akt (1:200, Proteintech, 10,176–2-AP, China), anti-pAkt (1:200, Cell Signaling Technology, 4060S, USA), or anti-Ki67 (1:100, Abcam, ab15580, UK) antibodies, incubated overnight at 4 °C and washed with PBS three times. Subsequently, sections were incubated with HRP-conjugated secondary antibody and streptavidin peroxidase. The average integral optical density of each positively stained section was measured by Image J software. Three random fields were selected for each section for measurement.

### Statistical Analysis

All experiments were performed in triplicate, and the results are presented as the mean value ± standard deviation (SD) from at least 3 biological replicates. The statistical significance of these data was analyzed by Student's t test or one-way ANOVA followed by GraphPad 8. Kaplan–Meier survival analysis and the log-rank test were used to analyze the overall survival of recurrent patients. Kaplan–Meier survival curves were used to assess the overall survival of patients and mice xenografts. A value of P < 0.05 was considered statistically significant. * indicates P < 0.05; ** indicates P < 0.01 and *** indicates P < 0.001.

## Results

### GSLC-Derived Exosomes Enhance GBM Cell Proliferation and Migration and Inhibit Glioblastoma Apoptosis

**Fig. 1:**
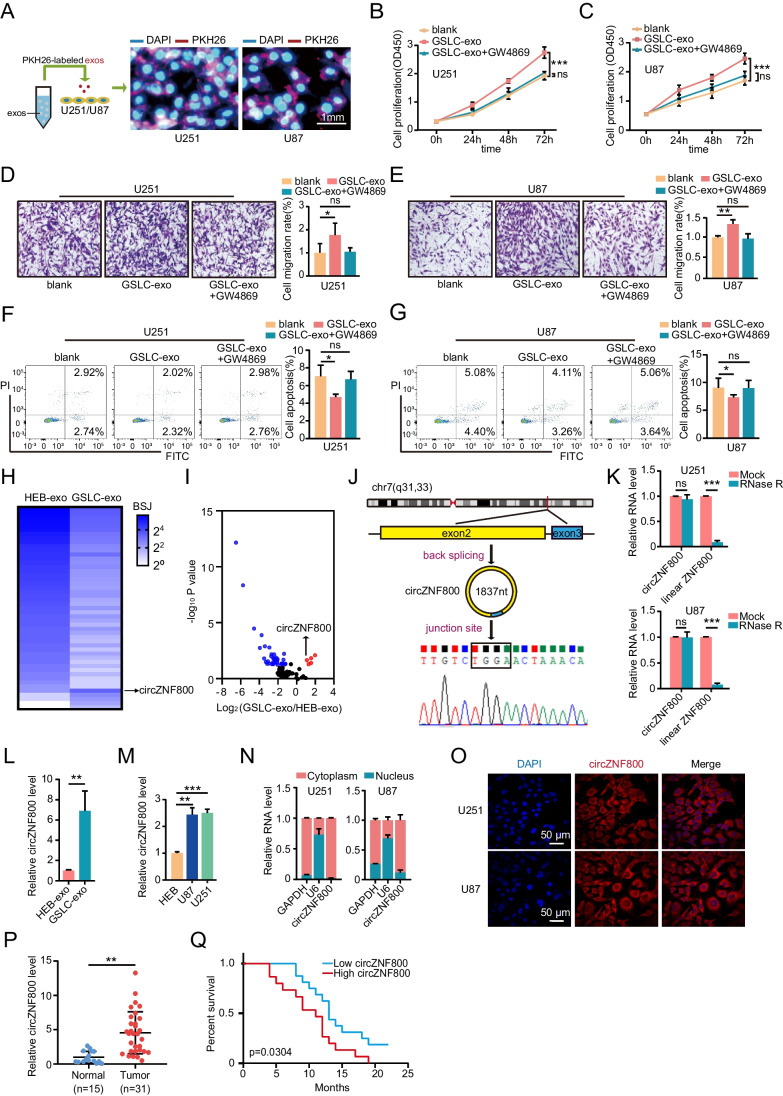
CircZNF800 was overexpressed in GSLC-derived exosomes and correlated with poor patient prognosis. **(A)** Exosomes from GSLCs were labeled with PKH26 and then added to U251 and U87 cell cultures (PKH26-red, DAPI-blue). Scale bar, 1 mm. **(B-C)** CCK-8 assay was used to evaluate the viability of U251 and U87 cells treated with GSLC-exos. GW4869 is applied to inhibit exosomes. **(D-E)** Transwell experiments measured the migration of U251 and U87 cells treated with GSLC-exos. GW4869 is applied to inhibit exosomes. **(F-G)** Flow cytometry assays measured the apoptosis ratio of U251 and U87 cells co-cultured with GSLC-exos. GW4869 is applied to inhibit exosomes. **(H)** Heatmap showing the differential expression of circZNF800 in the HEB-exos and GSLC-exos. **(I)** Volcano plots illustrating differential changes of circRNAs in HEB-exos versus GSLC-exos samples. Blue and red dots represent significantly down-regulated and up-regulated circRNAs, respectively **(J)** Schematic illustration indicating the generation of circZNF800 from its host gene and junction site validation by Sanger sequencing. **(K)** QRT-PCR analysis of circZNF800 and ZNF800 mRNA after treatment with or without RNase R in U251 and U87 cells. **(L)** QRT-PCR analysis of the expression of circZNF800 in HEB-exos and GSLC-exos. **(M)** The expression level of circZNF800 in HEB cells and glioblastoma cells (U87 and U251) was measured by qRT-PCR.** (N)** Cytoplasm and nuclear fractions were used to detect the location of circZNF800. GAPDH was used as a cytoplasmic negative control. U6 was used as a nuclear negative control. **(O)** The localization of circZNF800 was detected by RNA FISH in U87 and U251 cells. Nuclei were stained with 4,6-diamidino-2-phenylindole (DAPI). CircZNF800 was labeled with Cyanine 3 (Cyy3) dye. Scale bar, 50 μm **(P)** QRT-PCR assay detected the expression of circZNF800 in glioblastoma tissues (n = 31) compared to normal tissues (n = 15). GAPDH was used as a control. **(Q)** The 31 glioblastoma samples were divided into high and low groups based on circZNF800 expression. Kaplan–Meier survival curve analysis showed the relationship between the expression circZNF800 and glioblastoma patient survival. Data are presented as the mean ± S.D. The P value was determined by Student's t test or Kaplan–Meier survival curve analysis. Significant results are presented as NS nonsignificant, *P < 0.05, **P < 0.01, or ***P < 0.001

To explore cell communication in GBM microenvironments, we first isolated CD133 positive GSLCs from U87 cells and examined CD133 and Nestin levels, two markers for GSLCs, by Immunofluorescence assays (Supplementary Fig. 1A). To identify exosomes, we isolated exosomes from HEB cells and GSLCs, which were observed under transmission electron microscopy (Supplementary Fig. 1B). The nanoparticle size of HEB and GSLC exosomes was further measured by a nanoparticle analyzer (Supplementary Fig. 1C). Western blotting revealed the markers (CD9 and CD63) of exosomes, indicating the successful isolation of exosomes (Supplementary Fig. 1D). Subsequently, we examined whether these GSLC-derived exosomes (GSLC-exos) were taken up by GBM cells. These GSLC-exos were labeled with PKH26 (lipophilic fluorescent dye) and then added to GBM cells for co-culture (Fig. 1A). To investigate whether GSLCs affect GBM cells by secreting exosomes, GW4869 was applied for exosome inhibition in our work [27]. CCK-8 assay proved that GBM cell co-cultivation with GSLCs or GSLC-exos could enhance cell growth compared to that in the negative control groups (Supplementary Fig. 1E-F and Fig. 1B-C). Transwell assays revealed that U87 and U251 co-incubating GSLCs or GSLC-exos facilitated cell migratory capacity (Supplementary Fig. 1G-H and Fig. 1D-E). Flow cytometry validated that GBM cells co-cultured with GSLCs or GSLC-exos had a lower rate of apoptosis than negative control cells (Supplementary Fig. 1I-J and Fig. 1F-G). Taken together, these data might indicate that GSLCs regulate the tumorigenesis of GBM cells through exosomes.

### CircZNF800 is Highly Expressed in GSLC-exos and is Associated with Poor Prognosis in GBM Patients

Then, we asked how GSLC-derived exosomes regulate GBM tumorigenesis. Given that circRNAs are abundant in exosomes, we performed circRNA profiles from GSLC-exosomes and HEB-exosomes. We identified 49 differentially expressed circRNAs (fold change > 2 or < 0.5, P value < 0.05) in our dataset, among which 5 were upregulated and 44 were downregulated (Fig. 1H-I and Supplementary Fig. 1K).

We focused on hsa_circ_0082096, which is most significantly upregulated in GSLC-exos (Supplementary Fig. 1K). Hsa_circ_0082096, arising from the ZNF800 gene (we termed circZNF800) has a length of 1837 nt, which is located at chromosome 7 and consists of the head-to-tail splicing of exon 2 and exon 3. The putative back-splicing junction was confirmed by Sanger sequencing (Fig. 1J). The circular structure of circZNF800 was corroborated by the observed enrichment of circRNAs after RNase R treatment (Fig. 1K). QRT-PCR results revealed that circZNF800 was highly expressed in exosomes derived from GSLC compared with HEB exosomes (Fig. 1L). In addition, circZNF800 was higher in U251 and U87 cells than in HEB cells (Fig. 1M). RT-qPCR analysis of nuclear and cytoplasmic RNAs and fluorescence in situ hybridization with a probe against the back-spliced junction of circZNF800 were conducted, demonstrating that circZNF800 was preferentially localized within the cytoplasm in both U251 and U87 cells (Fig. 1N-O). Moreover, we detected the expression levels of circZNF800 in 31 GBM tissues (Table 1) and 15 normal tissues by qRT-PCR (Fig. 1P). In addition, we assessed the association between circZNF800 expression and prognosis in patients with GBM. Kaplan–Meier survival analysis revealed that patients with higher circZNF800 levels had a lower survival rate (Fig. 1Q). To summarize, circZNF800 was confirmed to be a circular RNA and was associated with poor prognosis in GBM patients.

### GLSC-exos CircZNF800 Promotes Malignant Progression of GBM Cells

**Fig. 2:**
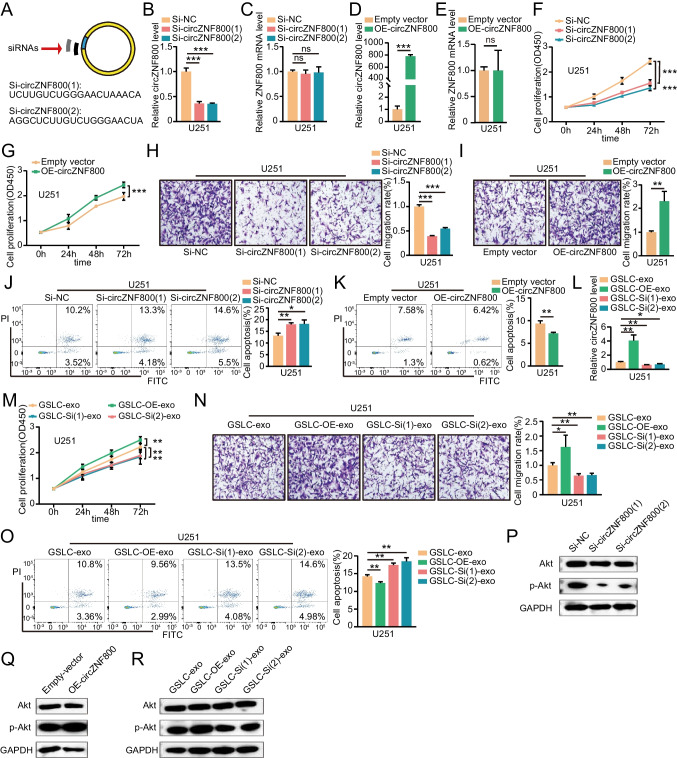
CircZNF800 promotes glioblastoma proliferation and migration and inhibits glioblastoma apoptosis in vitro. **(A)** Schematic diagram of the siRNA sequences specifically targeting the circZNF800 junction. **(B)** The expression of circZNF800 analyzed by qRT-PCR in Si-circZNF800 U251 cells. GAPDH was used as a control. **(C)** The expression of ZNF800 mRNA in U251 cells treated with two independent siRNAs. GAPDH was used as a control. **(D-E)** QRT-PCR verified the expression of circZNF800 and ZNF800 mRNA after transduction of OE-circZNF800 plasmids in U251 cells. GAPDH was used as a control.** (F-G)** CCK-8 assay analysis the effect of circZNF800 knockdown and overexpression on U251 proliferation. **(H-I)** Transwell assay tested the effect of circZNF800 knockdown and overexpression on U251 cell migration. **(J-K**) Annexin‐V FITC/PI staining detected the effect of circZNF800 knockdown and overexpression on U251 apoptosis. **(L)** QRT-PCR analysis of circZNF800 expression in U251 cells after treatment with exosomes derived from circZNF800-overexpressing GSLC (GSLC-OE-exo) or knockdown GSLC (GSLC-Si(1)-exo and GSLC-Si(2)-exo) cells. **(M)** The proliferation of U251 cells treated with various exosomes (GSLC-OE-exo, GSLC-Si(1)-exo and GSLC-Si(2)-exo) or control were detected by CCK-8 assay. **(N)** Transwell assays were used to detect the migration ability of U251 cells treated with various exosomes (GSLC-OE-exo, GSLC-Si(1)-exo and GSLC-Si(2)-exo) or control. **(O)** Flow cytometry detected the apoptosis of U251 cells treated with GSLC-exos, GSLC-OE-exos, GSLC-Si(1)-exos or GSLC-Si(2)-exos. **(P-R)** Western blotting analysis revealed that p-Akt activation was regulated by circZNF800. U251 cells transfecting with **(P)** si-circZNF800(1) or si-circZNF800(2), **(Q)** overexpression circZNF800, **(R)** GSLC-OE-exos, GSLC-Si(1)-exos or GSLC-Si(2)-exos. Data are presented as the mean ± S.D. The P value was determined by Student's t test. Significant results are presented as NS nonsignificant, *P < 0.05, **P < 0.01, or ***P < 0.001

To investigate the regulatory roles of circZNF800 in GBM cells, we performed loss-of-function analyses. Two independent siRNAs targeting the junction site of circZNF800 were used to knock down circZNF800 (Fig. 2A). The knockdown efficiency was validated by qRT-PCR, which showed that both siRNAs led to the significantly decreased expression level of circZNF800 (Fig. 2B and Supplementary Fig. 2A), but the level of ZNF800 mRNA was not affected (Fig. 2C and Supplementary Fig. 2B). Meanwhile, the qRT-PCR assay showed that circZNF800 could be upregulated when transfected with the overexpression of circZNF800 plasmid, but the level of ZNF800 mRNA was not affected (Fig. 2D-E and Supplementary Fig. 2C-D). Moreover, CCK-8 assay revealed that circZNF800 knockdown inhibited GBM cell proliferation, while circZNF800 overexpression facilitated GBM cell proliferation (Fig. 2F-G and Supplementary Fig. 2E-F). Transwell assays demonstrated that knockdown of circZNF800 could suppress GBM cell migration viability and overexpression of circZNF800 could promote GBM cell migration viability (Fig. 2H-I and Supplementary Fig. 2G-H). Flow cytometry revealed that knockdown and overexpression of circZNF800 could regulate cell apoptosis (Fig. 2J-K and Supplementary Fig. 2I-J). To further illustrate the functions of GLSC-exos circZNF800, we isolated exosomes from GSLCs transfected with two siRNAs against circZNF800 and overexpression of circZNF800 plasmids, (namely, GSLC-Si-exos and GSLC-OE-exos). We performed qRT-PCR to verify the expression of circZNF800 in these exos (Fig. 2L and Supplementary Fig. 2 K) and then added these exos to U251 and U87 cells for the next investigation. The CCK-8 assay indicated that GSLC-OE-exos could significantly promote the proliferation of GBM cells, but GSLC-Si-exos had the opposite effect (Fig. 2M and Supplementary Fig. 2L). The migration ability of the U251 and U87 cells was prominently increased by GSLC-OE-exos and decreased by GSLC-Si-exos which were confirmed by the transwell assay (Fig. 2N and Supplementary Fig. 2 M). Furthermore, the decline in apoptosis ratio was caused by GSLC-OE-exos compared with the control group. Instead, the increased ratio of apoptosis was due to GSLC-Si-exos (Fig. 2O and Supplementary Fig. 2N). Emerging studies revealed that circRNA/PI3K/Akt axis positively or negatively regulated the expression of tumor-relative genes and tumor progression, such as endometrial cancer and breast cancer [28–30]. Moreover, we then employed GSE153692 database [23, 25] to analyze the PI3K-Akt-related signaling in GBM. We found that PI3K-Akt-related mRNAs were activated in GBM patients. Thereby, we detected the relationship between circZNF800 and the activation of p-Akt by western blotting after GBM cells treated with si-circZNF800 or circZNF800. Western blotting revealed that circZNF800 silencing decreased AKT phosphorylation (p-Akt) in GBM cells (Fig. 2P and Supplementary Fig. 2O), whereas overexpressing circZNF800 increased AKT phosphorylation (Fig. 2Q and Supplementary Fig. 2P). To investigate whether GLSC-exos circZNF800 regulated activated AKT phosphorylation in GBM cells, we co-cultured GSLC-Si-exos and GSLC-OE-exos with GBM cells. Western blotting assays indicated that GSLC-OE-exos increased p-Akt, but GSLC-Si-exos had the opposite effects (Fig. 2R and Supplementary Fig. 2Q). Altogether, these results suggested that exosomal circZNF800 behaves as an oncogene in glioblastoma cells.

### CircZNF800 Serves as a miRNA Sponge of miR-139-5p

**Fig. 3:**
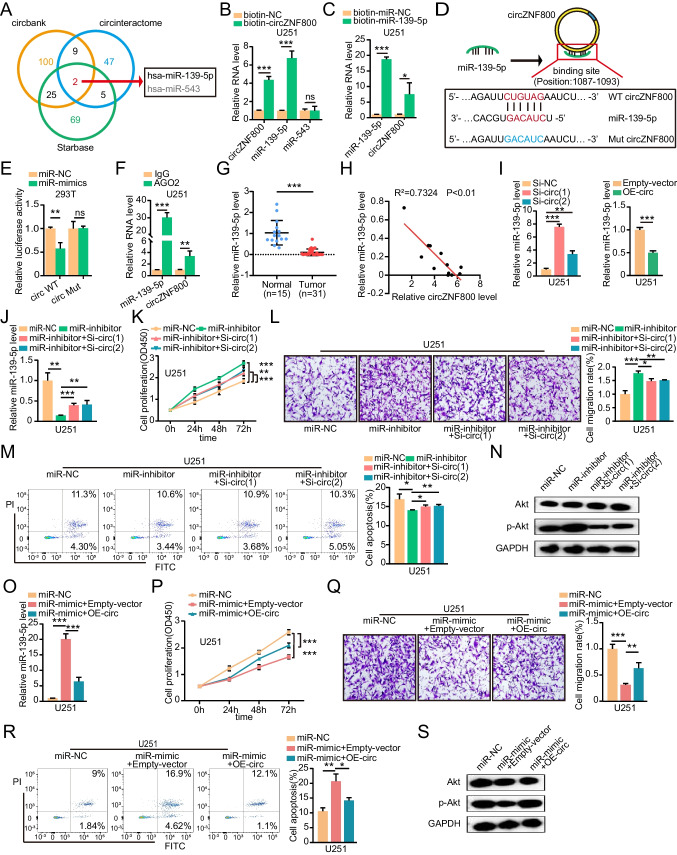
CircZNF800 functions as a sponge of miR-139-5p. **(A)** Venn diagram showing targets of circZNF800 predicted from circbank, circinteractome and StarBase. **(B)** Relative levels of circZNF800, miR-139-5p and miR-543 in U251 lysates were captured by the biotinylated probe circZNF800. GAPDH and U6 were used as controls. **(C)** Relative levels of circZNF800 and miR-139-5p in U251 lysates captured by the biotinylated probe of miR-139-5p. **(D)** Schematic of circZNF800 wild-type (WT) and mutant (Mut) luciferase reporter vectors. **(E)** Luciferase reporter gene assay to detect the interaction between circZNF800 and miR-139-5p. **(F)** RIP experiments were carried out in U251 cell extracts using an anti-AGO2 antibody. **(G)** The expression of miR-139-5p in GBM tissues and normal tissues by using qRT-PCR. U6 was used as a control. **(H)** Pearson correlation analysis of circZNF800 and miR-139-5p expression in GBM tissues (n = 12). **(I)** Expression level of miR-139-5p in U251 cells after transfection with si-circZNF800(1), si-circZNF800(2), or OE-circZNF800. **(J)** QRT-PCR was used to verify the efficiency of the miR-139-5p inhibitor was rescued by Si-circZNF800(1) and Si-circZNF800(2). **(K-M)** Detecting cell proliferation, migration and ratio of apoptosis of U251 treated miR-139-5p inhibitor alone or Si-circZNF800(1) and Si-circZNF800(2) respectively. **(K)** CCK-8 assays measured the migration ability of transfected U251 cells. **(L)** Transwell assays measured the migration ability of transfected U251 cells. **(M)** The apoptosis of treated U251 cells was detected by flow cytometry. **(N)** Western blotting was used to verify the activation level of p-Akt. **(O)** miR-139-5p expression in U251 cells transfected with miR-139-5p mimic alone or co-transfected with the OE-circZNF800 plasmid. **(P)** CCK-8 assay measured U251 cells transfected with miR-139-5p mimic alone or co-transfected with OE-circZNF800. **(Q)** The migration analysis of U251 cells transfected with miR-139-5p mimic alone or co-transfected with the OE-circZNF800 plasmid. **(R)** Annexin‐V FITC/PI staining was used to assess the apoptotic rates of U251 cells transfected with miR-139-5p mimic alone or co-transfected with the OE-circZNF800 plasmid. **(S)** The activation level of p-Akt was verified by western blotting. U251 cells transfected with miR-139-5p mimic or co-transfecting with the OE-circZNF800 plasmid. GAPDH was used as a control. Data are presented as the mean ± S.D. The P value was determined by Student's t test. Significant results are presented as NS nonsignificant, *P < 0.05, **P < 0.01, or ***P < 0.001

Many researchers have reported that circRNA assumes the role of a miRNA sponge in tumor development regulation [12, 16, 31]. To elucidate the molecular mechanism of circZNF800, we first predicted the targets of circZNF800 according to three databases circbank, circinteractome, and StarBase, and the results showed that 2 candidate miRNAs bond to circZNF800 (Fig. 3A and Table S2). Next, circZNF800 and miR-139-5p were captured and enriched by the biotin-labeled circZNF800 probe. On the other hand, miR-543 was not pulled down by circZNF800 probe, which showed that circZNF800 couldn't sponge miR-543 (Fig. 3B and Supplementary Fig. 3A). In addition, miR-139-5p and circZNF800 were pulled down by the biotin-coupled miR-139-5p probe in both GBM cell lines (Fig. 3C and Supplementary Fig. 3B). The binding sites (circZNF800-WT) and corresponding mutation sites (circZNF800-Mut) between circZNF800 and miR-139-5p are shown in Fig. 3D. To confirm that miR-139-5p bond to circZNF800 directly, a dual-luciferase reporter assay was performed. The luciferase activity of circZNF800-WT was reduced after co-transfection of the miR-139-5p mimic, but the luciferase activity of circZNF800-Mut did not change, which suggested that miR-139-5p was a target of circZNF800 in a sequence-specific manner (Fig. 3E). RNA immunoprecipitation (RIP) assays with an anti-AGO2 antibody were conducted in U251 and U87 cells to confirm whether circZNF800 acted as a miR-139-5p sponge in these cells, and the results showed that circZNF800 and miR-139-5p were enriched (Fig. 3F and Supplementary Fig. 3C). These findings indicated that circZNF800 acted as a miR-139-5p sponge. In addition, we confirmed that miR-139-5p was significantly reduced in GBM tissues compared to normal brain tissues (Fig. 3G). Moreover, miR-139-5p expression was negatively correlated with the expression of circZNF800 (Fig. 3H). QRT-PCR analysis showed that the expression of miR-139-5p was obviously increased after transfection with circZNF800 siRNAs in GBM cells while the overexpression of circZNF800 had the opposite effect, which further verified the interaction between circZNF800 and miR-139-5p (Fig. 3I and Supplementary Fig. 3D). We found that the miR-139-5p inhibitor suppressed the expression of miR-139-5p, but the effect of the miR-139-5p inhibitor could be rescued by knocking down circZNF800 (Fig. 3J and Supplementary Fig. 3E). To further investigate the roles of circZNF800 and miR-139-5p in GBM progression, we performed rescue assays to evaluate the effects of the circZNF800/miR-139-5p axis on the proliferation, migration and apoptosis abilities of GBM cells. CCK-8 results showed that the miR-139-5p inhibitor promoting cell growth was blocked by knocking down circZNF800 in GBM cells (Fig. 3K and Supplementary Fig. 3F). Similarly, transwell assay showed that the miR-139-5p inhibitor significantly boosted the invasion of U251 and U87 cells. However, the knockdown of circZNF800 eliminated these effects (Fig. 3L and Supplementary Fig. 3G). Flow cytometry showed that the miR-139-5p inhibitor markedly inhibited apoptosis of GBM cells, while knockdown of circZNF800 eliminated this effect (Fig. 3M and Supplementary Fig. 3H). As shown in Fig. 3N, p-Akt protein was remarkably promoted by the miR-139-5p inhibitor, whereas the level of p-Akt was retarded by si-circZNF800 (Fig. 3N and Supplementary Fig. 3I). In the same way, the high expression of miR-139-5p induced by miR-139-5p mimic was reversed by overexpression of circZNF800 (Fig. 3O and Supplementary Fig. 3J). The CCK-8 assay showed that the inhibition of cell proliferation by miR-139-5p mimic was alleviated by overexpression of circZNF800 (Fig. 3P and Supplementary Fig. 3K). The promotion of apoptosis by miR-139-5p mimic was moderated by overexpression of circZNF800 (Fig. 3R and Supplementary Fig. 3M). We found that the expression of p-Akt was markedly downregulated in the miR-139-5p mimic group, while after transfecting circZNF800, the expression of these proteins was upregulated (Fig. 3S and Supplementary Fig. 3N). These results suggested that circZNF800 regulates the proliferation, migration and apoptosis of GBM cells by sponging miR-139-5p.

### PIEZO1 is a Direct Target of miR-139-5p and is Regulated by CircZNF800

**Fig. 4:**
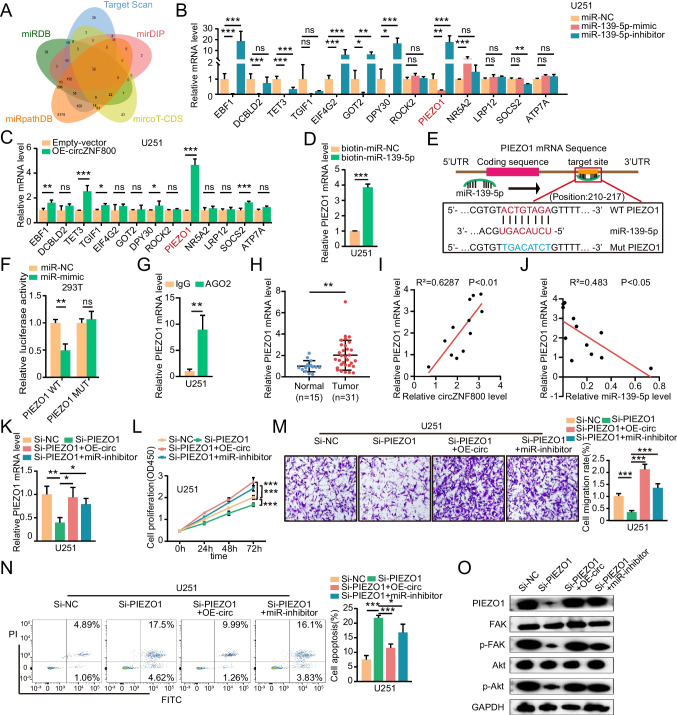
PIEZO1 is a direct target of miR-139-5p and is regulated by circZNF800. **(A)** The target genes of miR-139-5p predicted by TargetScan, miRDB, miRpathBD, microT-CDS, and miRDIP. **(B-C)** The mRNA expression levels of downstream genes targeted by miR-139-5p were measured by qRT-PCR in U251 cells treated with **(B)** miR-139-5p mimic or miR-139-5p inhibitor, **(C)** OE-circZNF800. **(D)** The RNA pulldown assay was performed with relative levels of PIEZO1 in U251 lysates captured by the biotinylated probe of miR-139-5p. **(E)** The schematic diagram shows the binding site of miR-139-5p and the PIEZO1 3'UTR. **(F)** Luciferase reporter gene assay to detect the interaction between circZNF800 and miR-139-5p. **(G)** The RIP assay detected the expression of PIEZO1 mRNA in U251 cell lysates using an anti-AGO2 antibody. IgG antibody was used as a control. **(H)** The expression of PIEZO1 mRNA in glioblastoma (n = 31) tissues and normal tissues (n = 15) was measured by qRT-PCR. **(I)** Pearson correlation analysis of PIEZO1 mRNA and circZNF800 expression in glioblastoma tissues (n = 12). **(J)** Pearson correlation analysis of PIEZO1 mRNA and miR-139-5p expression in glioblastoma tissues (n = 12). **(K)** The qRT-PCR assay detected the expression of PIEZO1 mRNA in U251 cells after transfection with Si-PIEZO1 alone or co-transfection with OE-circZNF800 or miR-139-5p inhibitor. **(L)** CCK-8 assay was performed to assess U251 cell growth ability after transfection with Si-PIEZO1 alone or co-transfection with OE-circZNF800 or miR-139-5p inhibitor. **(M)** Transwell assays were performed to assess the migration ability of U251 cells after transfection with Si-PIEZO1 alone or co-transfection with OE-circZNF800 or miR-139-5p inhibitor. **(N)** Flow cytometry analysis showed the apoptosis of U251 cells after transfection with Si-PIEZO1 alone or co-transfected with OE-circZNF800 or miR-139-5p inhibitor. **(O)** Western blotting analysis of the protein levels of PIEZO1, FAK, p-FAK, Akt and p-Akt in U251 cells. Data are presented as the mean ± S.D. The P value was determined by Student's t test. Significant results are presented as NS nonsignificant, *P < 0.05, **P < 0.01, or ***P < 0.001

The potential target genes of miR-139-5p were predicted by five miRNA target gene databases TargetScan, miRDB, mirDI, microT-CDS, miRpathDB (Fig. 4A and Table S3). The overlap of five miRNA target gene databases showed 32 alternative mRNAs, but according to the GEPIA and CGGA databases, only 13 candidate mRNAs were upregulated in GBM patients. To determine whether miR-139-5p could regulate the expression of downstream targets, we examined the expression of these mRNAs by qRT-PCR after knocking down or overexpressing miR-139-5p. PIEZO1 mRNA expression was most significantly increased by miR-139-5p inhibition, while PIEZO1 mRNA expression was significantly decreased by the miR-139-5p mimic in GBM cells (Fig. 4B and Supplementary Fig. 4A). Compared with the control group, overexpression of circZNF800 significantly increased the mRNA expression of PIEZO1 (Fig. 4C and Supplementary Fig. 4B). Previous study has suggested that PIEZO1 is overexpressed in cell membrane, and its expression is negatively correlated with human aggressive glioma patients survival [32]. To confirm the interaction of miR-139-5p and PIEZO1 mRNA, we performed an RNA pulldown assay of miR-139-5p and found that miR-139-5p was able to pulldown PIEZO1 mRNA (Fig. 4D and Supplementary Fig. 4C). To further elucidate the molecular mechanism by which miR-139-5p regulated PIEZO1 expression, we discovered that the PIEZO1 mRNA 3'UTR contains a potential miR-139-5p binding site (Fig. 4E). The results of the dual-luciferase reporter assay demonstrated that the relative luciferase activity of the PIEZO1 wild-type significantly decreased, suggesting that PIEZO1 was a target of miR-139-5p (Fig. 4F). In the RIP assay, PIEZO1 mRNA was enriched in the miRNA ribonucleoprotein complex containing AGO2 compared with control IgG (Fig. 4G and Supplementary Fig. 4D). Additionally, the expression of PIEZO1 mRNA was higher in GBM tissues than in normal tissues (Fig. 4H). Correlation analysis showed that the expression levels of circZNF800 and PIEZO1 mRNA were positively correlated (Fig. 4I), and the expression levels of miR-139-5p and PIEZO1 mRNA were negatively correlated in GBM tissues from clinical patients (Fig. 4J). To evaluate the function of PIEZO1 in GBM, we designed a siRNA-targeted PIEZO1 mRNA coding sequence. CircZNF800 or miR-139-5p significantly attenuated the effects of PIEZO1 siRNA (Fig. 4K and Supplementary Fig. 4E). We analyzed cell proliferation, migration and apoptosis and found that PIEZO1 deletion reduced proliferation (Fig. 4L and Supplementary Fig. 4F) and migration (Fig. 4M and Supplementary Fig. 4G) and promoted apoptosis (Fig. 4N and Supplementary Fig. 4H) in U251 and U87 cells, but these effects were rescued by circZNF800 or miR-139-5p inhibitor (Fig. 4L-N, Supplementary Fig. 4F-H). A study revealed that PIEZO1 could upregulate the expression of p-Akt by activating focal adhesion kinase (FAK) [33]. As a non-receptor tyrosine kinase, FAK is an important signaling component that is activated by a variety of stimuli and acts as a biosensor to control cell movements, including proliferation and migration. FAK autophosphorylates at Y397 could additionally recruit members of the Src-family of kinases (SFKs). The proximity of SFKs to FAK is thought to lead to the activation of downstream effectors, including Akt. Previous reports have highlighted phosphorylation of FAK at Y397 as an important regulator of the angiogenic response in vitro [34–36]. Our western blotting results demonstrated the ascending protein expression of PIEZO1, the activated FAK and activated Akt transfected siPIEZO1 alone or co-transfected with circZNF800 or miR-139-5p inhibitor (Fig. 4O and Supplementary Fig. 4I). In order to illustrate circZNF800 could regulates the progression of GBM by affecting PIEZO1 and increasing intracellular Ca2 + signaling, we conducted calcium imaging experiments and found that decreasing Ca2 + signaling when silencing circZNF800 (Supplementary Fig. 4J). Taken together, these results revealed that circZNF800 regulates GBM cell proliferation, migration and apoptosis via the miR-139-5p/PIEZO1/Akt axis.

### Silencing CircZNF800 can Inhibit Glioblastoma Growth and Metastasis in Vivo

**Fig. 5:**
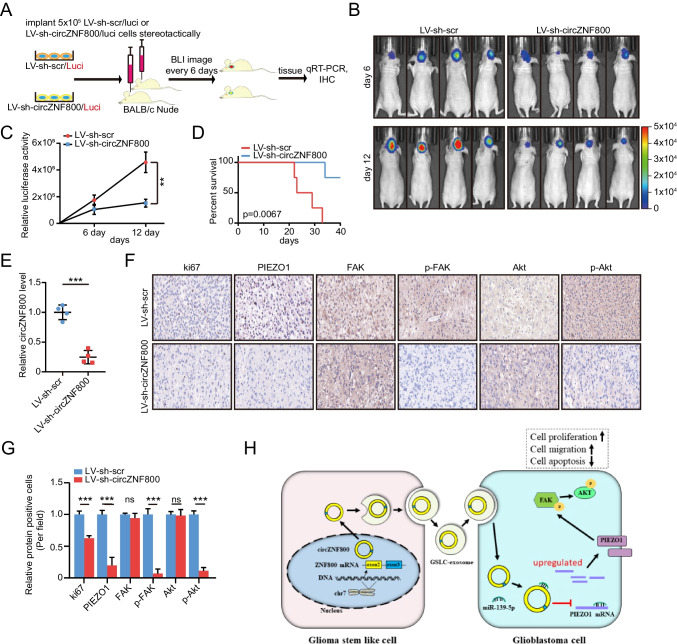
Silencing circZNF800 can inhibit glioblastoma growth and metastasis in vivo. **(A)** The schematic diagram shows LV-sh-scr/luci and LV-sh-circZNF800/luci U251 cells (5 × 10^5^ cells) orthotopic xenotransplantation in nude mice. **(B)** Bioluminescent images of nude mice. **(C)** Quantitative study of bioluminescence imaging signal intensity in nude mice. **(D)** Kaplan–Meier curve analysis showed the survival of xenograft models between LV-sh-scr and LV-sh-circZNF800 group. **(E)** The expression of circZNF800 was measured by qRT-PCR assay in xenograft tumors. **(F)** The expression of Ki-67, PIEZO1, FAK, p-FAK, Akt and p-Akt was examined by immunohistochemistry in xenograft tumors (400 ×). **(G)** Relative proteins (Ki-67, PIEZO1, FAK, p-FAK, Akt and p-Akt) positive cells in LV-sh-scr or LV-sh-circZNF800 cell-derived tissues were analyzed by IHC. **(H)** The schematic diagram shows how circZNF800 derived from GSLCs could promote tumorigenesis of GBM through circZNF800/PIEZO1/Akt axis. Data are presented as the mean ± S.D. The P value was determined by Student's t test or Kaplan–Meier survival curve analysis. Significant results are presented as NS nonsignificant, **P < 0.01, or ***P < 0.001

To evaluate the function of circZNF800 in vivo, we established U251 stable cell line with lentivirus to knockdown circZNF800. The procedure of in vivo xenografts assay was showed in Fig. 5A. Bioluminescence imaging revealed that knockdown of circZNF800 reduced the malignant GBM xenografts to the negative control group (Fig. 5B). We found that when circZNF800 was knocked down, the luciferase activity of the tumor was dramatically increased (Fig. 5C), while overall survival was decreased compared with that of the control group (Fig. 5D). Moreover, our qRT-PCR analysis suggested that circZNF800 was downregulated in GBM xenograft models (Fig. 5E). In addition, immunohistochemistry (IHC) of resected tumor sections showed the expression of Ki67, PIEZO1, FAK, p-FAK, Akt and p-Akt in tumor tissues with or without circZNF800 knockdown (Fig. 5F). Finally, we determined knocking down circZNF800 could decrease the expressions of PIEZO1, Ki67, p-FAK and p-Akt in tumor tissues (Fig. 5G). Taken together, our data suggested that circZNF800 played an important role in regulating GBM progression and tumorigenesis.

## Discussion

Previous studies have shown that glioblastoma (GBM) is challenging for neurosurgeons because of its rapid proliferation and invasive growth, which can invade normal brain tissue and lead to incomplete resection and recurrence [37]. Glioma stem-like cells (GSLCs) exhibit stem-like properties and are thought to be responsible for the high recurrence rate of GBM [38–40]. GSLCs are small populations of cells in glioma tumor samples with stem-cell associated characteristics, such as self-renewal ability, expression of stem-cell markers, and differentiate into multiple nervous system lineages (neurons, astrocytes, and oligodendrocytes) [41, 42]. Related researches reported that GSLCs promoted GBM tumorigenic phenotype by regulating molecular networks, for example, the cytokine receptor for oncostatin Moncostatin M (OSMR) improves glioblastoma response to ionizing radiation [5]. Another example of GSLCs in a hypoxic environment regulated glioblastoma chemoresistance by upregulating JAG1 and DLL4 [43]. Therefore, a therapeutic strategy to target GSLCs has become an choice to suppress GBM development [44].

Studies of extracellular vesicles can help identify unknown cellular and molecular mechanisms involved in intercellular communications and diseases [10]. Exosomes are a subset of EVs with an average diameter of ~ 100 nm. Nucleic acids, proteins, and lipids are selectively bound to intraluminal vesicles, which are located in multivesicular endosomes and are precursors of exosomes [45]. Exosomes have been proven to be important information exchange carriers between cells, including GSLCs and GBM [46, 47]. Evidence from Xiong et al. suggested that *linc01060* gene derived from hypoxic glioma stem cell exosomes can directly interact with the transcription factor myeloid zinc finger 1 (MZF1) to promote MZF1-mediated c-Myc transcriptional activity, thereby leading to glioma progression [47]. Our previous research revealed that glioma stem-like cells derived exosomal miR-155-5p may regulate mesenchymal transition by directly targeting ACOT12 and enhance the invasiveness of glioma [7]. In a word, exosomes provide a window into the altered state of glioma cells or tissues, and their detection in biological fluids may provide a multicomponent diagnostic reference.

Circular RNAs (circRNAs) are a class of non-coding RNAs, with a covalently closed circular structure [14]. With the rapid development of high-throughput sequencing, emerging studies have shown that circRNAs in exosomes derived from tumor cells play an important role in tumor formation, angiogenesis, invasion, metabolic reprogram, and chemotherapy resistance[13, 48–50]. For example, *circRNA_104797* was upregulated in sorafenib resistant hepatocellular carcinoma and was essential for the maintenance and spread of sorafenib resistance in HCC. Mechanically, *circRNA_104797* which was propagated via exosomes, interacteed with YBX1 in the cytoplasm to prevent PRP19-mediated YBX1 ubiquitination and degradation in the nucleus [13]. In addition, *circCARM1* served breast cancer stem cell exosomes as vectors to regulate breast cancer cell metabolic reprogramming through the miR-1252-5p/PFKFB2 pathway [50]. However, studies on glioma stem-like cell derived exosomal circRNAs regulating the malignant phenotype and mechanism of glioblastoma have not been clearly reported.

MicroRNAs (miRNAs), with an average nucleotide of ~ 21 nt, are evolutionarily conserved and are encoded in the genomes of almost all eukaryotes. MiRNAs participate in post-translation biological progress, especially in animals, by base pairing to partially complementary sequences in the 3'untranslated regions (UTRs) of target mRNA [51]. Researches demonstrated that miR-139-5p served as a tumor suppressor by regulating tumor-related proteins in colorectal cancer, gastric cancer and glioblastoma [52–54]. In our research, we verified that miR-139-5p was downregulated in glioblastoma tissues and suppressed glioblastoma cell lines deterioration, which was consistent with previous research results.

PIEZO1 is known as an ion channel that allows the permeation of cations, including sodium, potassium, and calcium, which is reported to function as a homeostatic role in heart health, innate immunity and vascular development [55–57]. A recent study reported that PIEZO promoted glioma tissue stiffening and tumor cell proliferation, which provided a strategy for targeting PIEZO1 to break the disease-aggravating feedforward circuit [32].

Our present study revealed that exosomes from GSLCs could enhance the migration of GBM cells, suggesting that exosomes played a regulatory role in the GBM malignant phenotype. In this study, we identified a novel circRNA (hsa_circ_0082096) from GSLC-exos, namely, circZNF800. The expression of circZNF800 was positively correlated with GBM patient survival. Using a loss/gain of function methods, we demonstrated that exosomal circZNF800 regulated proliferation, migration and apoptosis in GBM. Moreover, our results indicated that circZNF800 was distributed predominantly in the cytoplasm and served as a microRNA sponge. Mechanistically, we found that GSLC-derived exosomal circZNF800 regulated PIEZO1/FAK/Akt signaling by sponging to miR-139-5p (Fig. 5H). Most importantly, we confirmed that knockdown of circZNF800 significantly inhibited GBM proliferation and migration in xenograft mouse models. Our study identified that circZNF800 and miR-139-5p are upstream of PIEZO1, which might provide genetic targets for the treatment of glioblastoma. In a word, our study may provide novel insights into the mechanisms involved in GBM progression. We found circZNF800 from GSLC exosomes and revealed that exosomal circZNF800 plays an important role in regulating the miR-139-5p/PIEZO1/FAK/Akt signaling axis.

### Supplementary Information

Below is the link to the electronic supplementary material.

Supplementary file1 (PDF 1585 KB)

Supplementary file2 (DOCX 24 KB)

Supplementary file3 (XLSX 13 KB)

Supplementary file4 (XLSX 108 KB)

Supplementary file5 (PDF 6809 KB)

## Data Availability

Quantitative data that support this study are available within this article and its supplementary files. All other data that support the fundings of this study are available from the corresponding author on reasonable request.
