# Supplementary figures and images for: Exosomal circZNF800 Derived from Glioma Stem-like Cells Regulates Glioblastoma Tumorigenicity via the PIEZO1/Akt Axis
Source: Mol Neurobiol. 2024 Feb 7;61(9):6556–71. doi: 10.1007/s12035-024-04002-0 (PMC11338982; doi:10.1007/s12035-024-04002-0)

# Supplementary Figure 1

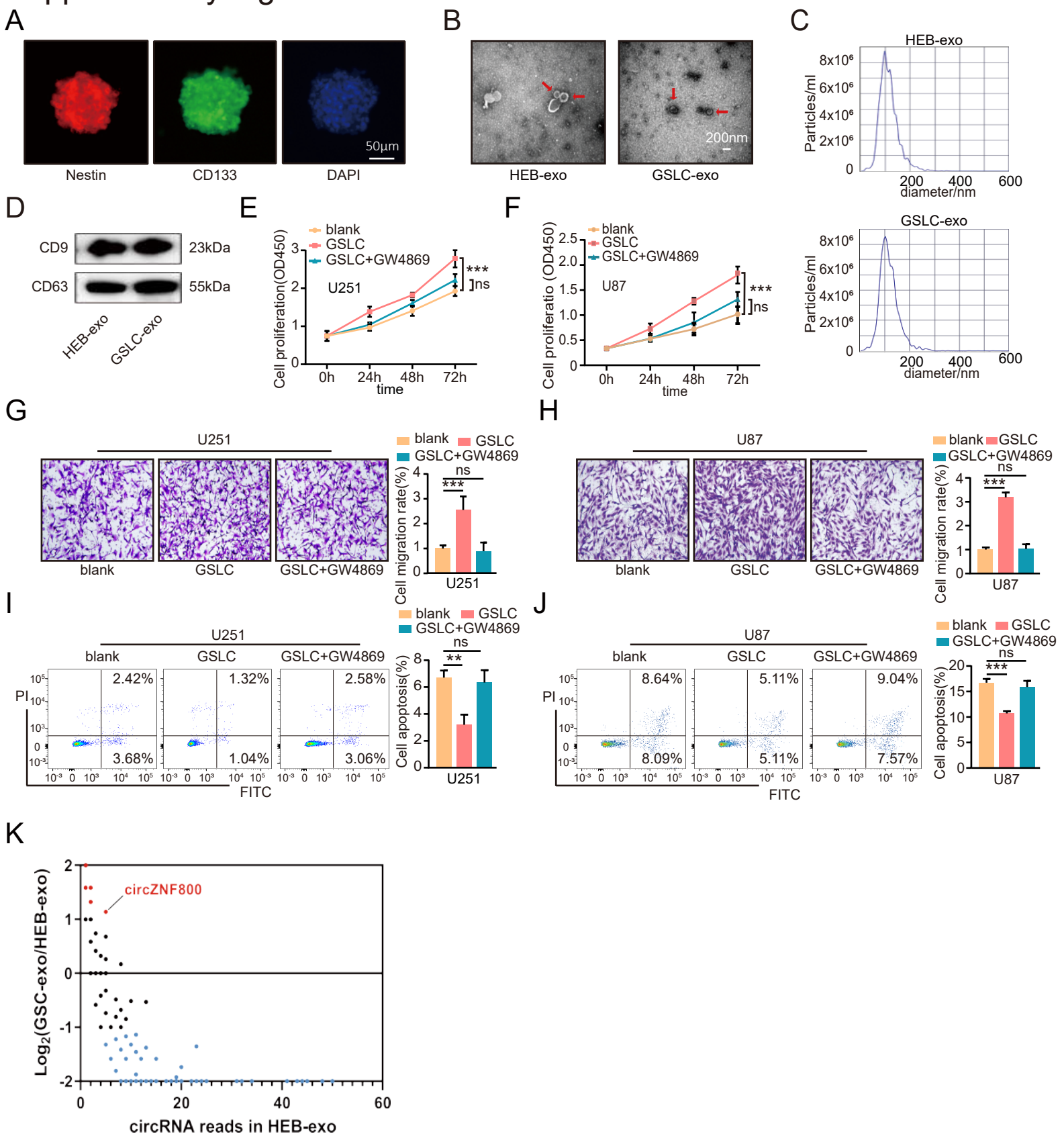

# Supplementary Figure 2

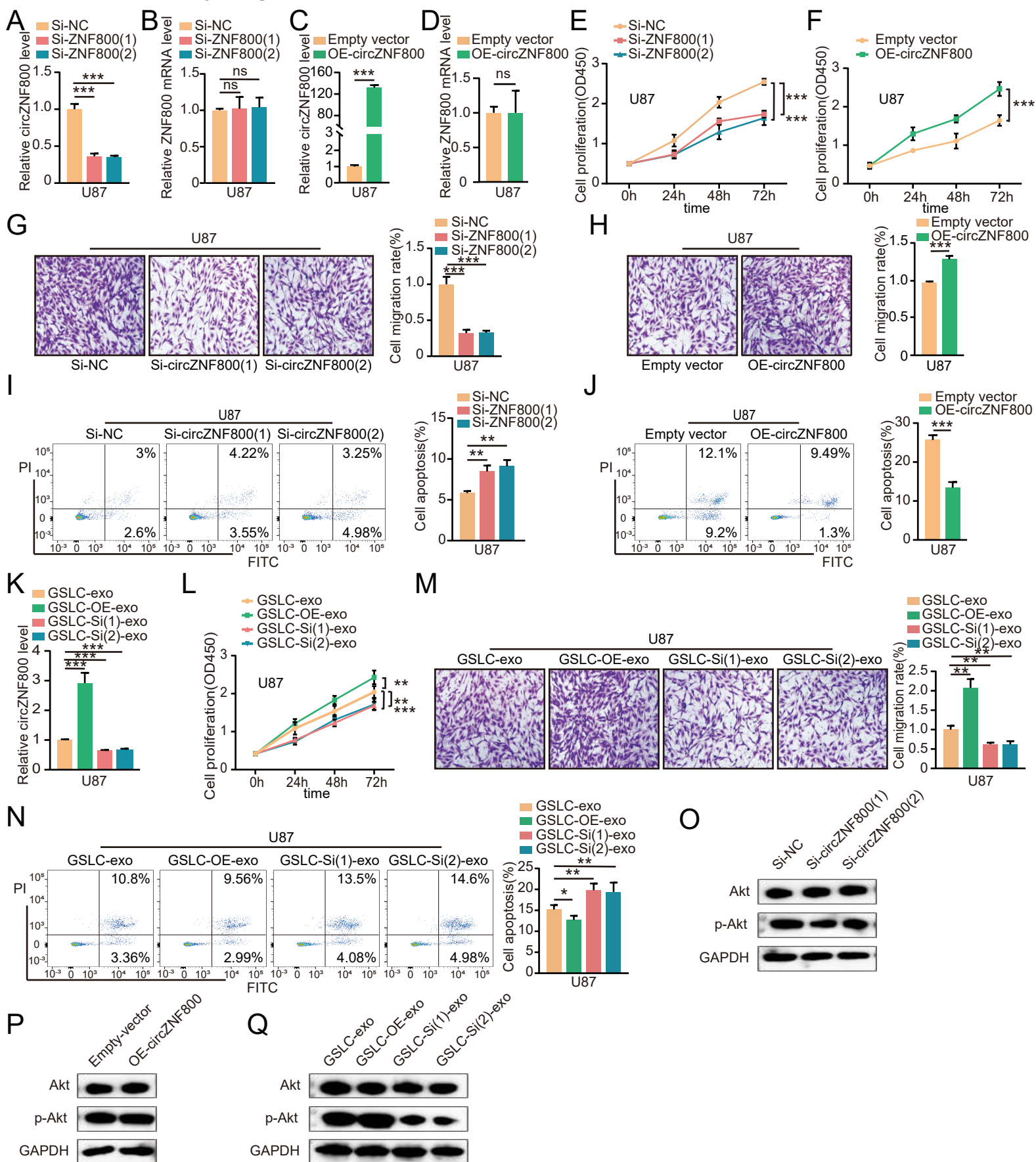

# Supplementary Figure 3

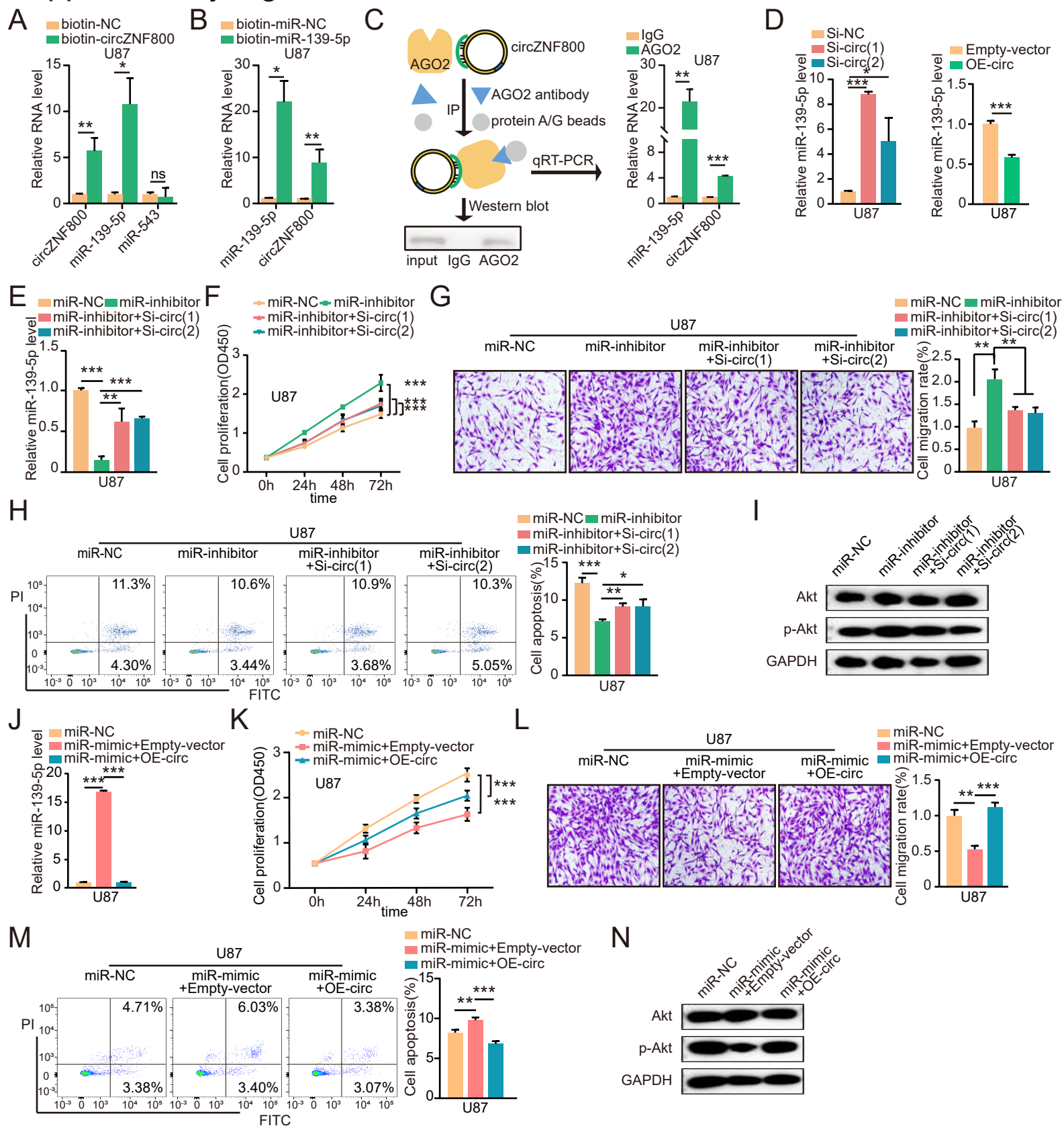

# Supplementary Figure 4

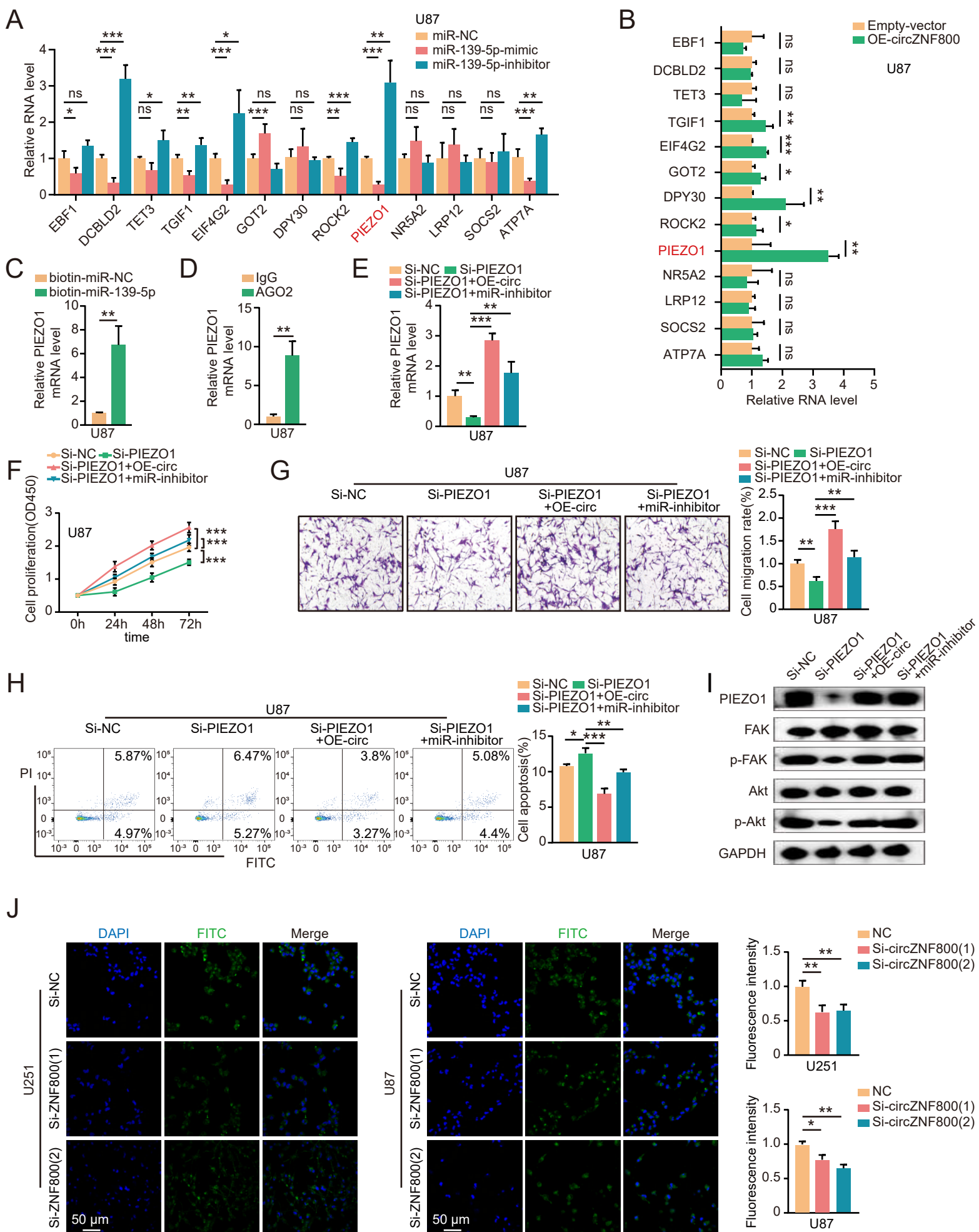

Supplement: Supplementary file 1 — Supplementary file1 (PDF 1585 KB) [file 12035_2024_4002_MOESM1_ESM.pdf]
