# Supplementary material for: Exosomal circZNF800 Derived from Glioma Stem-like Cells Regulates Glioblastoma Tumorigenicity via the PIEZO1/Akt Axis
Source: Mol Neurobiol. 2024 Feb 7;61(9):6556–71. doi: 10.1007/s12035-024-04002-0 (PMC11338982; doi:10.1007/s12035-024-04002-0)
Supplement: Supplementary file 2 — Supplementary file2 (DOCX 24 KB) [file 12035_2024_4002_MOESM2_ESM.docx]

**Supplementary Table 1 Oligos used in the study**

| has-circZNF800-F | AAGGCCGAAGTACAAGATCT | For circZNF800  qRT-PCR |
| --- | --- | --- |
| has-circZNF800-R | ATGTAAGTTATGCCGCAGTC |  |
| miR-139-5p-F | ACACTCCAGCTGCACGTGTC | For miR-139-5p  RT-PCR |
| miR-139-5p-R | TGGTGTCGTGGAGTCGGTTGA |  |
| miR-543-F | CCAGCTACACTGGGCAGCA GCAATTCATGTTT | For miR-543  RT-PCR |
| miR-543-R | CTCAACTGGTGTCGTGGA |  |
| GAPDH-F | CTTCATTGACCTCAACTACATGG | For GAPDH qRT-PCR |
| GAPDH-R | CTCGCTCCTGGAAGATGGTGAT |  |
| U6-F | CGCTTCGGCAGCACATATAC | For U6 qRT-PCR |
| U6-R | TTCACGAATTTGCGTGTCAT |  |
| PIEZO1-F | CTCTTCCTGGCGCTGTTC | For PIEZO1 qRT-RCR |
| PIEZO1-R | GATGAGGTTGGTGGAGTTGG |  |
| EBF1-F | AAAGCATCCAACGGAGTGGAA | For EBF1 qRT-PCR |
| EBF1-R | GCCCTGTCTGTCGTAGAGG |  |
| DCBLD2-F | ATGTGGACACACTGTACTAGGC | For DCBLD2 qRT-PCR |
| DCBLD2-R | CTGTTGGGATAGGTCTGTGGG |  |
| TET3-F | GCCGGTCAATGGTGCTAGAG | For TET3 qRT-PCR |
| TET3-R | CGGTTGAAGGTTTCATAGAGCC |  |
| TGIF1-F | GGGATTGGCTGTATGAGCACC | For TGIF1 qRT-PCR |
| TGIF1-R | GGCGGGAAATTGTGAACTGA |  |
| EIF4G2-F | GGGGTGCTTCTCGTTTCAGT | For EIF4G2 qRT-PCR |
| EIF4G2-R | AGCAGTCTTGGGATAGTGCTG |  |
| GOT2-F | AAGAGGGACACCAATAGCAAAAA | For GOT2 qRT-PCR |
| GOT2-R | GCAGAACGTAAGGCTTTCCAT |  |
| DPY30-F | GGAGGGACAAACGCAGGTT | For DPY30 qRT-PCR |
| DPY30-R | GGTAGGCACGAGTTGGCAA |  |
| ROCK2-F | TCAGAGGTCTACAGATGAAGGC | For ROCK2 qRT-PCR |
| ROCK2-R | CCAGGGGCTATTGGCAAAGG |  |
| NR5A2-F | CTTTGTCCCGTGTGTGGAGAT | For NR5A2 qRT-PCR |
| NR5A2-R | GTCGGCCCTTACAGCTTCTA |  |
| LRP12-F | GTAGCTGGTTCATAAGGGCAAA | For LRP12 qRT-PCR |
| LRP12-R | CCAGATGTGGTCTTGTGAAGAGA |  |
| SOCS2-F | TTAAAAGAGGCACCAGAAGGAAC | For SOCS2 qRT-PCR |
| SOCS2-R | AGTCGATCAGATGAACCACACT |  |
| ATP7A-F | TGACCCTAAACTACAGACTCCAA | For ATP7A qRT-PCR |
| ATP7A-R | CGCCGTAACAGTCAGAAACAA |  |
| si-NC | UUCUCCGAACGUGUCACGU  ACGUGACACGUUCGGAGAA | Negative control |
| si-circZNF800-1 | UUGCUUUGAGGGAAGUUAC GUAACUUCCCUCAAAGCAATT | siRNAs of circZNF800 |
| si-circZNF800-2 | UUCCUUUUUCUGGAGAACG CGUUCUCCAGAAAAAGGAATT |  |
| si-PIEZO1 | CAUCAGAGGAGAACUUCGAUCGAAGUUCUCCUCUGAUG | siRNAs of PIEZO1 |
| circZNF800-probe1 | AGTTGTTTAGTTCCCAGACAAGAGCCTTAG | For circZNF800 RIP with 5’biotin labeled |
| circZNF800-probe2 | GCTTAAGTTGTTTAGTTCCCAGACAAGAGC |  |
| circZNF800-probe3 | TAAGTTGTTTAGTTCCCAGACAAGAGCCTT |  |
| Srcamble | TTCTCCGAACG-TGTCACGTTCGAACGTGTC | Control probe with 5’biotin labeled |
