# Supplementary material for: Exosomal circZNF800 Derived from Glioma Stem-like Cells Regulates Glioblastoma Tumorigenicity via the PIEZO1/Akt Axis
Source: Mol Neurobiol. 2024 Feb 7;61(9):6556–71. doi: 10.1007/s12035-024-04002-0 (PMC11338982; doi:10.1007/s12035-024-04002-0)

Figure 2 (P, Q, R)

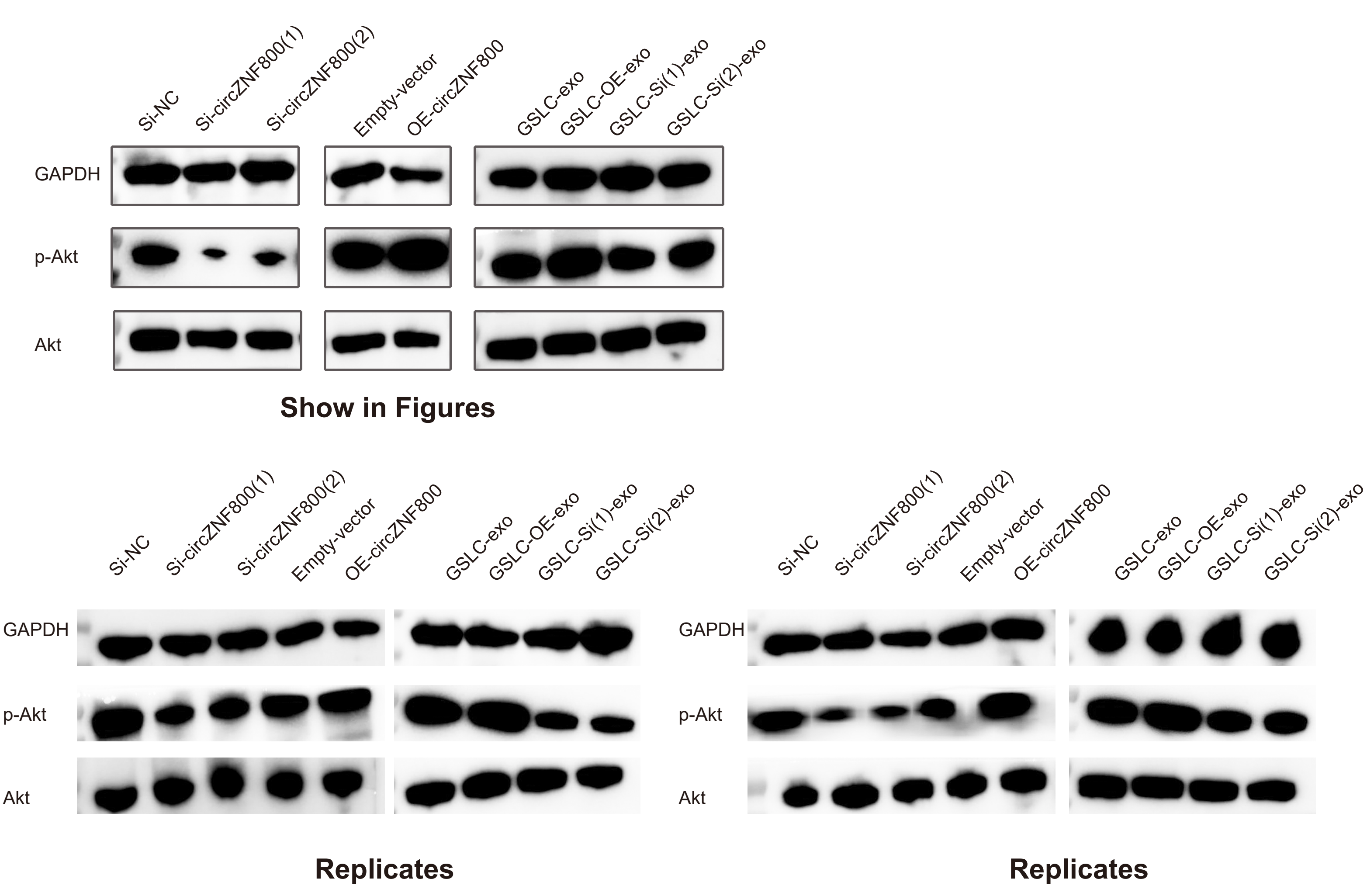

Figure 3 (N)

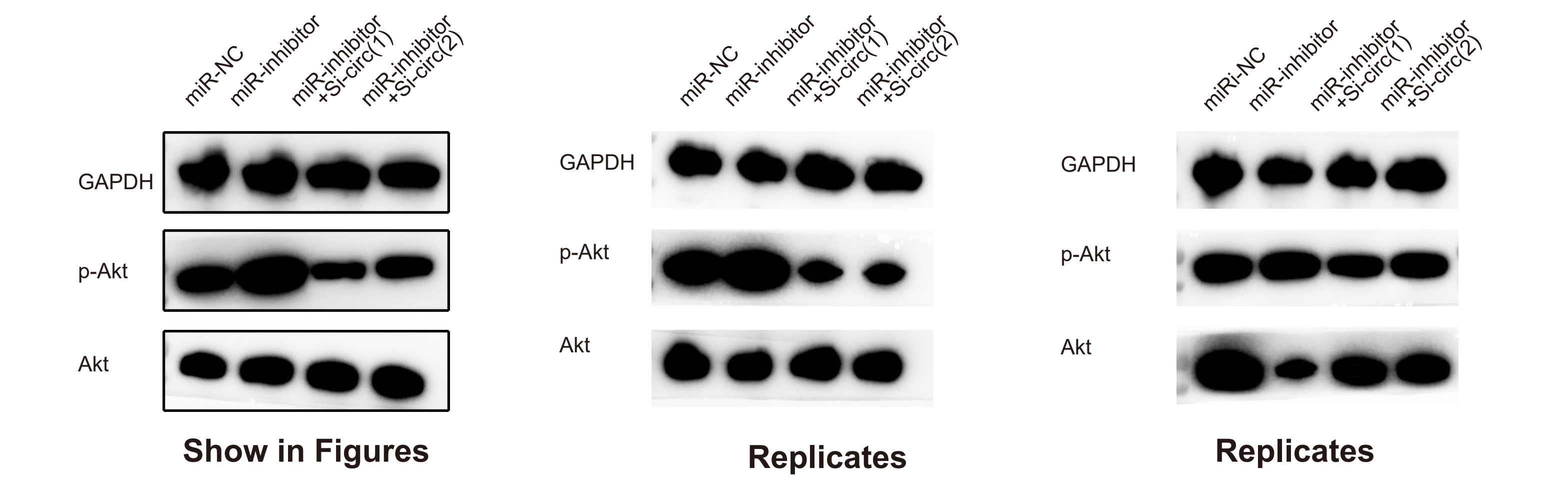

Figure 3 (S)

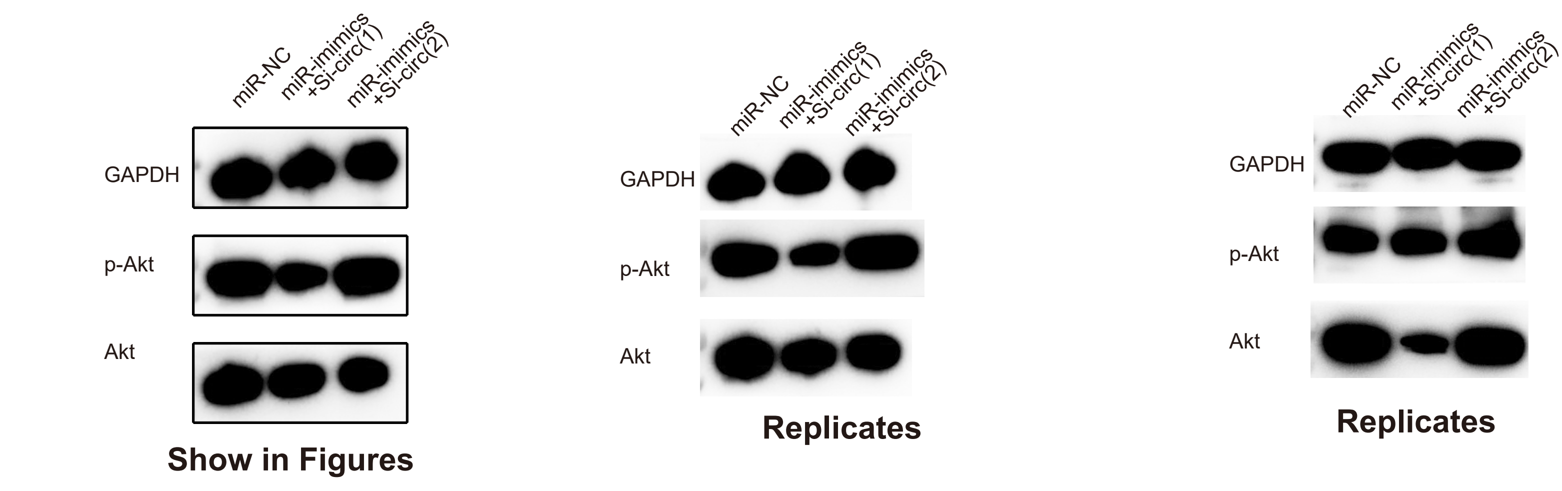

Figure 4 (O)

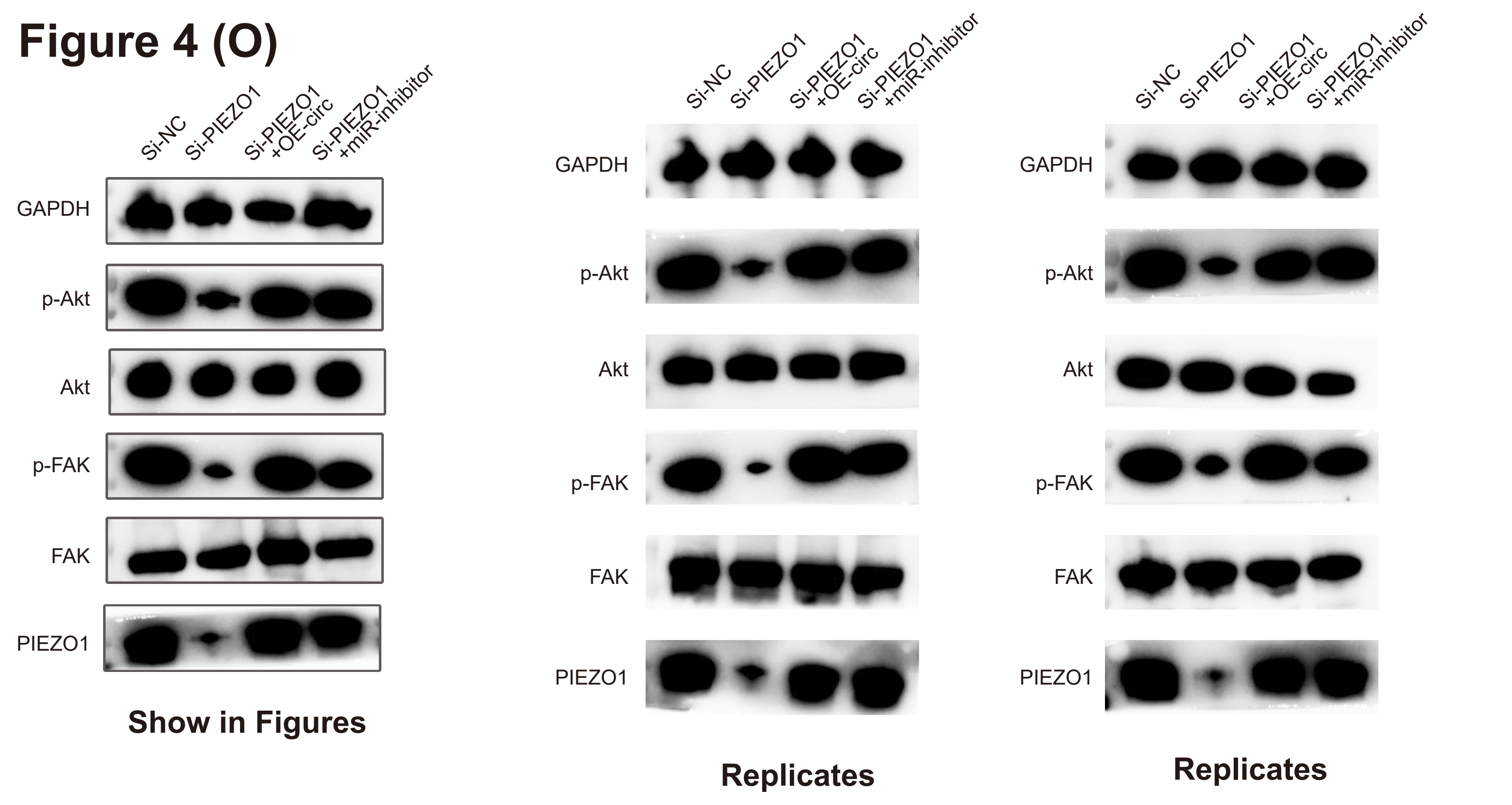

Supplementary Figure 2 (O, P, Q)

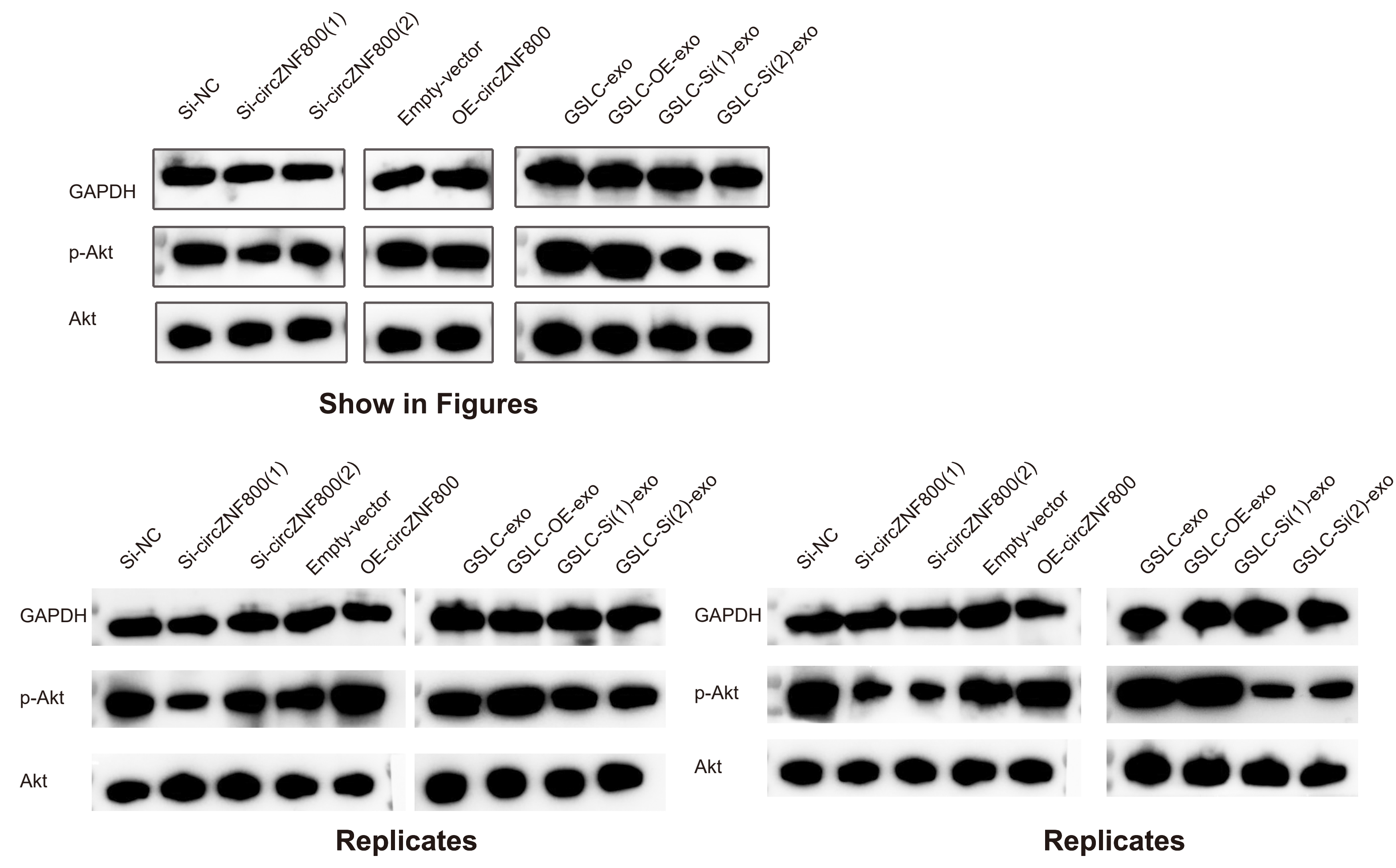

Supplementary Figure 3 (I)

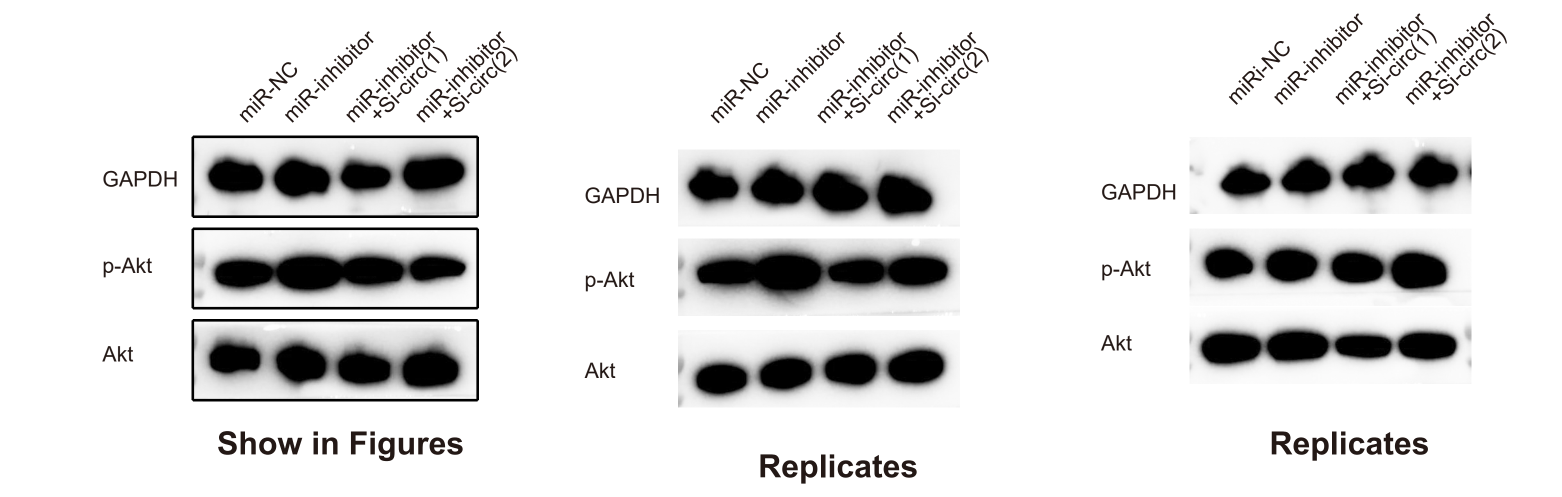

Supplementary Figure 3 (N)

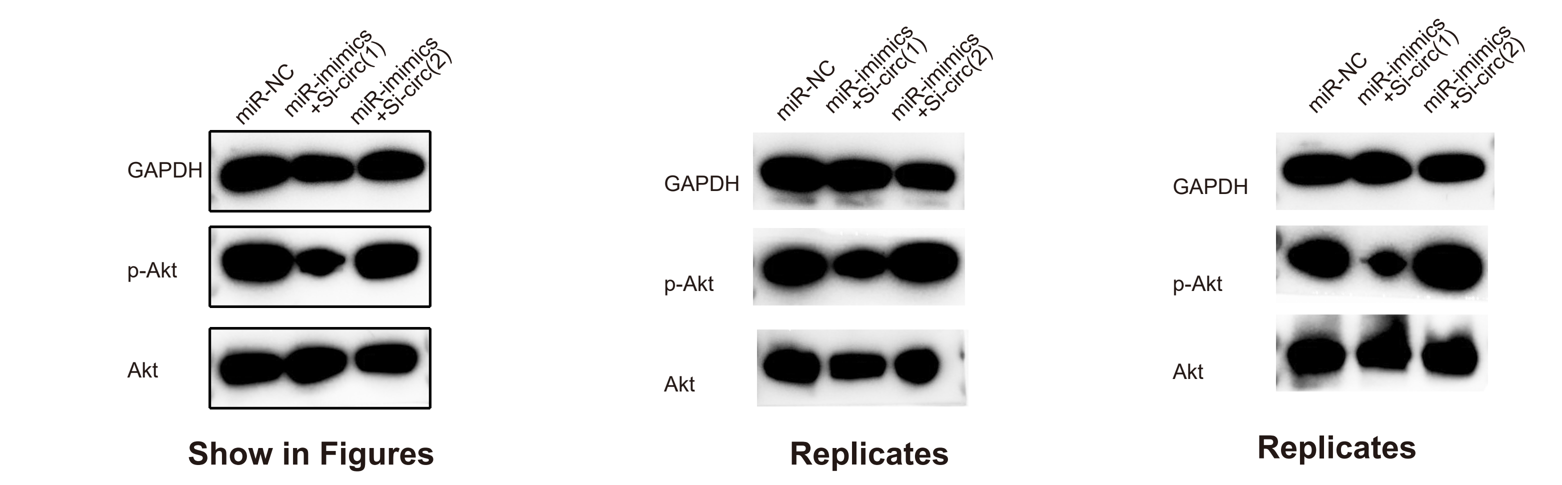

Supplementary Figure 4 (I)

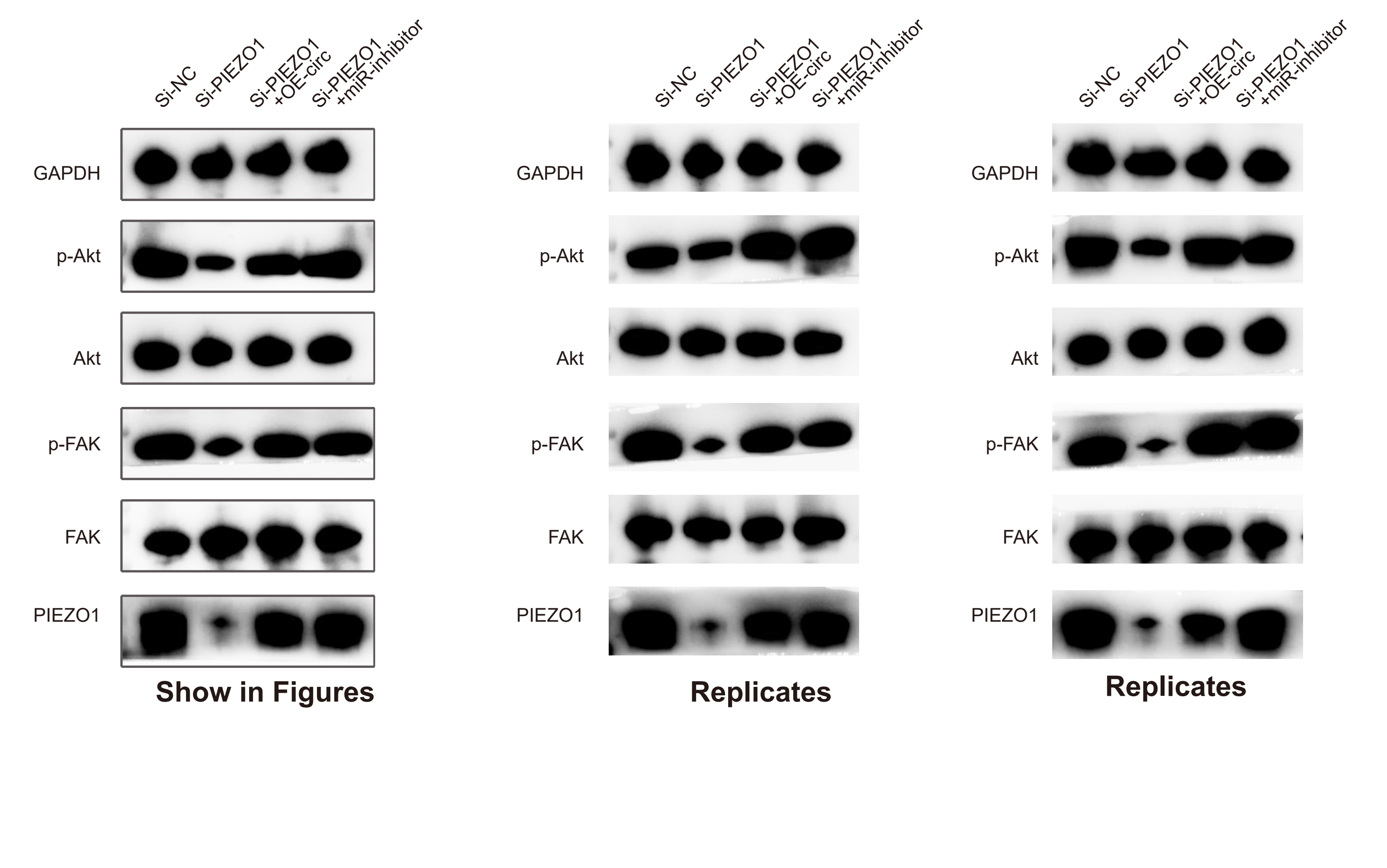

Supplement: Supplementary file 5 — Supplementary file5 (PDF 6809 KB) [file 12035_2024_4002_MOESM5_ESM.pdf]
